# Exogenous ABA and IAA modulate physiological and hormonal adaptation strategies in *Cleistocalyx operculatus and Syzygium jambos* under long-term waterlogging conditions

**DOI:** 10.1186/s12870-022-03888-z

**Published:** 2022-11-10

**Authors:** El-Hadji Malick Cisse, Juan Zhang, Da-Dong Li, Ling-Feng Miao, Li-Yan Yin, Fan Yang

**Affiliations:** 1 School of Ecological and Environmental Sciences, Hainan University, Haikou, 570228 China; 2 School of Life Sciences, Hainan University, Haikou, 570228 China; 3 School of Plant Protection, Hainan University, Haikou, 570228 China; 4 Key Laboratory of Agro-Forestry Environmental Processes and Ecological Regulation of Hainan Province, Center for Eco-Environmental Restoration Engineering of Hainan Province, Haikou, 570228 China

**Keywords:** ABA, Adventitious roots, GA_3_, Hormonal crosstalk, IAA, Jasmonic acid, Photosynthesis

## Abstract

**Background:**

The mechanisms of abscisic acid (ABA) and auxin (IAA) in inducing adventitious root (AR) formation, biomass accumulation, and plant development under long-term waterlogging (LT-WL) conditions are largely unexplored. This study aimed to determine the roles of exogenous application of ABA and IAA in two woody plants (*Cleistocalyx operculatus* and *Syzygium jambos*) under LT-WL conditions. A pot experiment was conducted using a complete randomized design with two factors: (i) LT-WL and (ii) application of exogenous phytohormones (ABA and IAA) for 120 d.

**Results:**

Results revealed that exogenous ABA and IAA promoted LT-WL tolerance in both species. In *C. operculatus* and *S. jambos*, plant height, the number of blades, leaf area, and fresh shoot weight were increased by exogenous IAA under LT-WL. However, exogenous ABA affected more the adventitious and primary root in *C. operculatus* compared to *S. jambos*. LT-WL decreased drastically the photosynthetic activities in both species, but adding moderate amounts of exogenous ABA or IAA protected the photosynthesis apparatus under LT-WL. Exogenous phytohormones at certain levels decreased the superoxide anion level and malondialdehyde accumulation in plants under LT-WL. Also, the increase of the peroxidases and superoxide dismutase activities by exogenous phytohormones was more marked in *C. operculatus* compared to *S. jambos*. Meanwhile, the catalase activity was down-regulated in both species by exogenous phytohormones. Exogenous ABA or IAA positively regulated the jasmonic acid content in ARs under LT-WL. Moderate application of exogenous ABA or IAA in plants under LT-WL decreased the ABA content in the leaves. Lower accumulation of IAA and ABA in the leaves of *C. operculatus* under LT-WL was positively correlated with a decrease in antioxidant activity.

**Conclusions:**

Lastly, *C. operculatus* which has greater morphology indexes was more tolerant to waterlogging than *S. jambos*. Moreover, the adaptive strategies via exogenous ABA were more built around the below-ground biomass indexes particularly in *C. operculatus*, while exogenous IAA backed the above-ground biomass in both species. Overall, the exogenous hormones applied (spraying or watering) influenced differentially the plant’s responses to LT-WL. The phytohormonal profile of plants exposed to waterlogging stress varied depending on the species’ tolerance level.

**Supplementary Information:**

The online version contains supplementary material available at 10.1186/s12870-022-03888-z.

## Background

Long-term waterlogging (LT-WL) is one of the vital environmental stresses that can drastically affect and limit the growth and distribution of plants. Waterlogging can reduce the gas exchange amid soil and air, resulting in 10,000-fold reduction in gas diffusion in water. Under submergence conditions, O_2_ in the soil rapidly decrease and the soil can become hypoxic or anoxic within few hours [1]. Waterlogging lead to soil nutrient deficiencies or toxicities, affects plant growth, and results in roots death, even though damage entire plant [2–4]. The waterlogging tolerant plant species have developed diverse and complex strategies to survive under waterlogging condition, including morphological, anatomical, and physiological adaptations as well as hormonal interactions [5, 6]. During waterlogging, numerous morphological changes occurred, such as the formation and development of adventitious roots (ARs). These types of roots are different from lateral or primary roots, as based on a general definition that ARs arise from non-root tissues [7]. ARs can be form from hypocotyl pericycle cells, phloem or xylem parenchyma cells, young secondary phloem cells, or interfascicular cambium cells close to the phloem cells [8]. Plant hormones, such as abscisic acid (ABA) and auxin (IAA), and photosynthesis are involved in the regulation and promotion of adventitious roots, which is a common adaptive response of plants to waterlogging. ABA is an important and a small organic signaling molecule synthesize in plant roots and shoots. ABA is involved in diverse mechanisms in plants, including germination, seed dormancy, root development, stress tolerance, stomatal closure, and growth [9]. Meanwhile, IAA plays prominent functions in plant growth and development by controlling cell division, elongation, and differentiation [10]. Previous studies focused on phytohormone-mediated plant growth and development under waterlogging conditions. It has been well established the crucial roles of IAA in plant growth and ARs formation under stress. However, the roles of ABA in ARs biomass accumulation and waterlogging tolerance via plant hormones and photosynthesis activity remain still ambiguous, but there are interesting indications that ABA takes part in ARs formation and waterlogging tolerance [11]. Indeed, recent published articles [12], [13], showed the prominent role of the leaves in ARs formation. Indeed, those reports revealed particularly that IAA-leaf act as a guru to manage ARs formation in which diverse transcription factors and mechanically sensitive microtubules are involved. Also, this report pointed out the crucial relation between IAA and other phytohormones such as jasmonates, cytokinins, strigolactones, and ethylene. However, interconnections between IAA-leaf, IAA-ARs, ABA-leaf, and ABA-ARs were out of the screen, despite the fact that the report displayed a detailed connection amongst ARs formation with the leaf photosynthetic activity through the carbohydrate variations. The central function of ABA is the plant abiotic stress tolerance through photosynthesis regulation [14], [15]. However, the knowledge of the crosstalk amid ABA and IAA accumulation in plant leaves and ARs with the photosynthesis is still incomplete, especially during waterlogging. In the present research work, particular attention is given to the phytohormone contents in the leaves and ARs of two tree species under waterlogging, to shed light on relationships between photosynthesis, leaf-IAA, leaf-ABA, ARs-IAA and ARs-ABA. It has been reported that ARs emergence in plants subjected to waterlogging was associated with an increase of IAA level and decrease of ABA level [16]. Moreover it has been suggested that ABA acts as a negative regulator of ARs formation under waterlogging as well as decrease in the ABA content of stem node in wheat [17]. However, an earlier study showed that ABA may stimulate ARs initiation in mung bean by regulating IAA and peroxidase activity [18]. Few studies have showed that ABA can maintain or promote root growth under stress conditions, which change our view of the role of ABA as a growth inhibitor plant hormone [19]. It is clear that the roles of ABA in root growth (stimulate/repress) depend on root type, environmental conditions, genotypes and species [16]. Thus, it is worthwhile to explore the role and interactions of phytohormones such as ABA or IAA in plants under LT-WL environments.

According to previous studies, exogenous IAA increased drought tolerance in *Trifolium repens* by activating auxin, ABA, and jasmonic acid (JA-Me) related genes and suppressing senescence genes [20]. Moreover, IAA application in *Withania somnifera* mitigated the effects of supplemental ultraviolet-B radiation by reducing the over-accumulation of reactive oxygen species (ROS) via an increase in the antioxidant activities [21]. It has been reported that Indole-3-butyric acid (IBA) uphold adventitious rooting in *Arabidopsis* via its conversion into indole-3-acetic acid and stimulation of anthranilate synthase activity [22]. Exogenous IAA can also mitigate copper toxicity in *Spinacia oleracea* by an increase of the antioxidant activities and nitrogen metabolism [23]. Meanwhile, exogenous ABA applied in the roots of *Zea mays* improved chilling tolerance [24]. ABA applied in *Atractylodes macrocephala* minimized lead-induced toxicity via the antioxidant system [25]. Moreover, it has been well established the crucial roles of exogenous ABA in the regulation of root and cell hydraulic conductivity and abundance of some aquaporin in *Hordeum vulgare* [26].

Numerous studies have been conducted to investigate the application of exogenous IAA by spray or chemigation to alleviate abiotic stresses [20], [21], [23]. The spraying of ABA has been used in various plants to increase their stress tolerance [25]. The uses of exogenous IAA and ABA improve waterlogging tolerance in plants, on the other hand, is not a common practice. For example, chemigation was an unusual use of exogenous ABA in plants to improve waterlogging tolerance [6], [27]. Moreover, the last decades have witnessed the emergence of several studies that supported the transport of exogenous IAA and ABA from the shoot to the roots and vice versa [6], [28–30], thus different ways used in the present study may provide some glows on how IAA and ABA affect plant growth and endogenous plant hormone under stress following their spraying or chemigation in two woody plants.

*Cleistocalyx operculatus* and *Syzygium jambos*, belong to Myrtaceae family, are medicinal woody plants with certain waterlogging tolerance [31], [32]. Also, it is relevant to notice that the responses of both species to environmental stresses are quasi-inexistent in literature. *S. jambos* is a shrub commonly known as a rose apple which is widespread in Southeast Asia. *S. jambos* is well known for its medicinal properties and used for a variety of ailments [33], [34]. Concurrently, *S. jambos* and *C. operculatus* are well-known medicinal plants and their buds are commonly used as an ingredient in tonic drinks in China [35]. Thus, the present study aimed to provide a support of the roles of ABA and IAA in LT-WL conditions in two trees species that might display different adaptations strategies to waterlogging via adventitious biomass accumulation, increase photosynthesis efficiency, and boost antioxidant system. Furthermore, based on literatures cited above, we speculate that exogenous ABA might promote waterlogging tolerance in both species via the root system and antioxidant system. While IAA may mitigate waterlogging-induced damages and promote plant growth via ARs formation and development, and antioxidant machinery.

## Results

### Plant growth evaluation

**Fig. 1:**
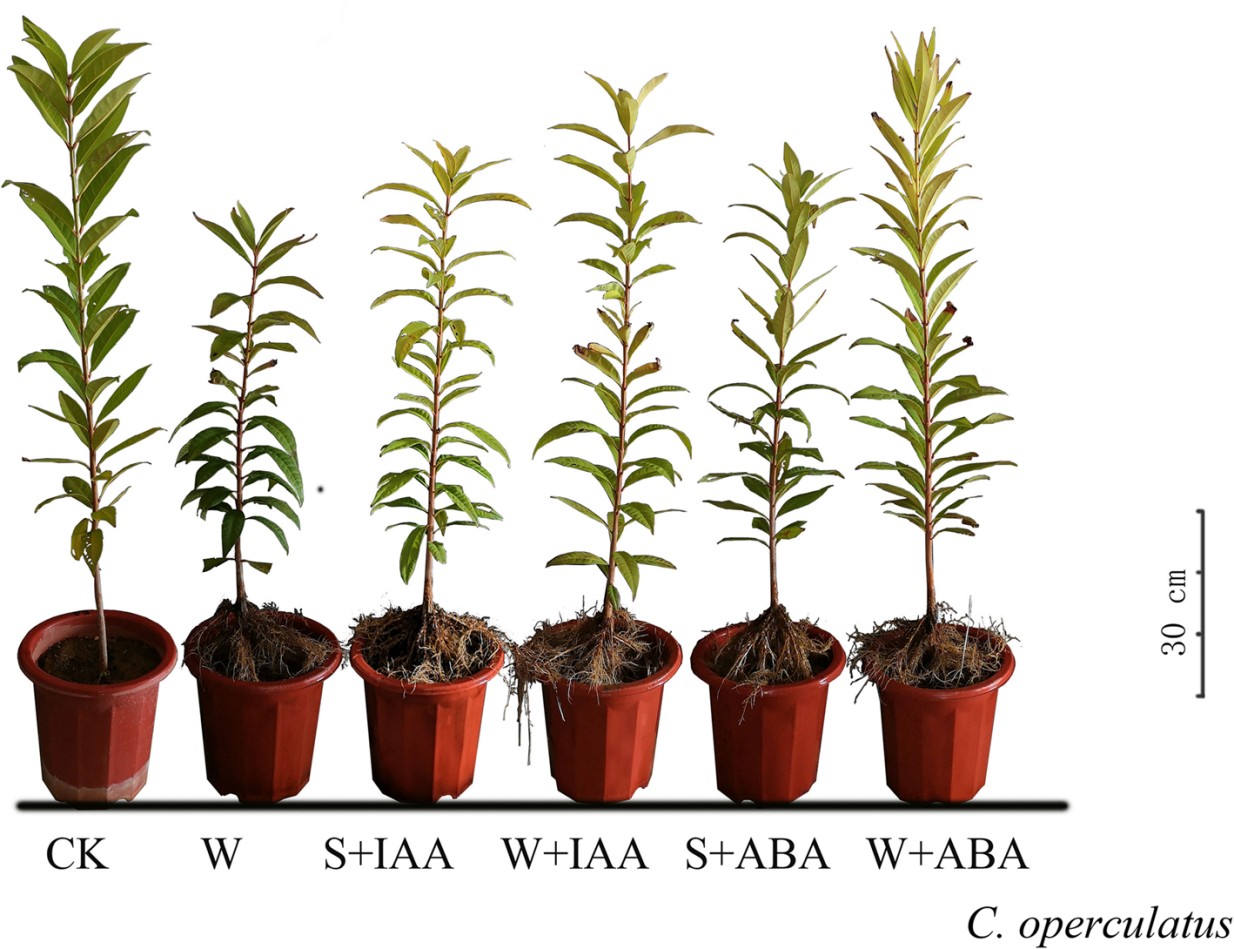
Morphological appearance of *C. operculatus* under LT-WL and subjected to exogenous IAA and ABA

**Fig. 2:**
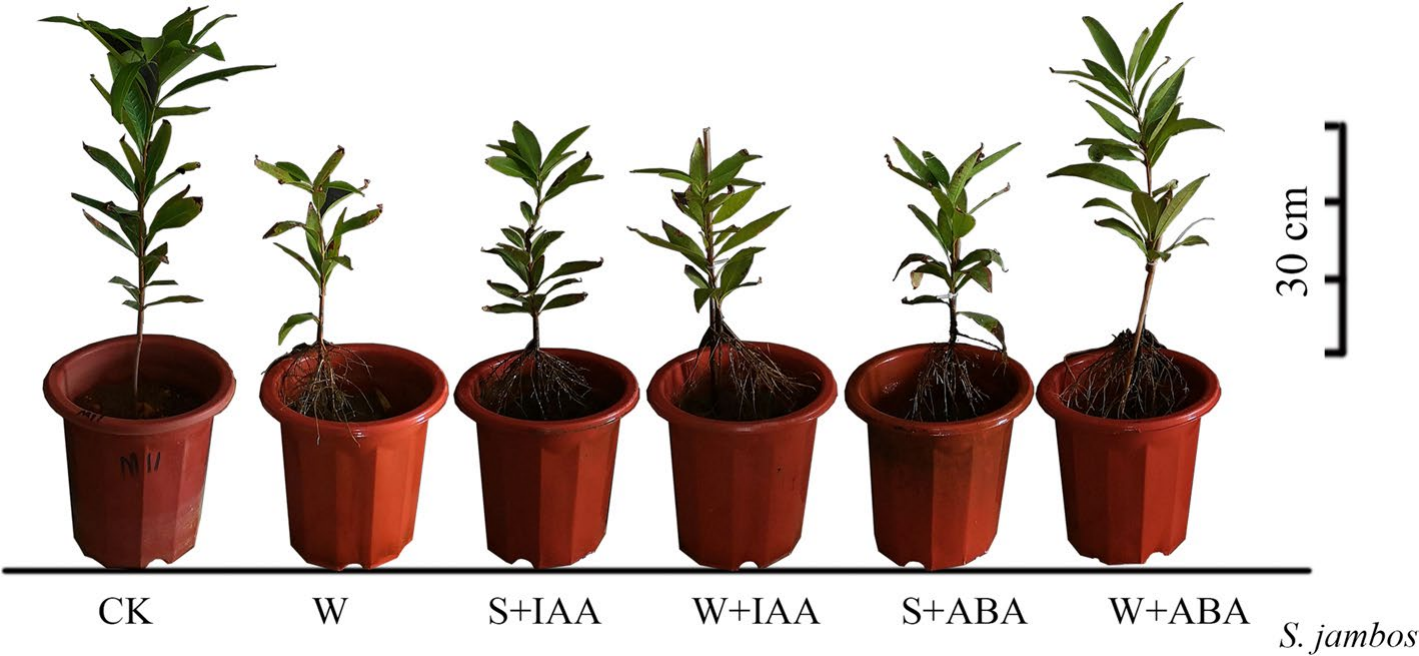
Morphological appearance of *S. jambos* under LT-WL and subjected to exogenous IAA and ABA

In response to waterlogging (WL), *C. operculatus* and *S. jambos* showed a significant decrease in biomass accumulation but an increase in adventitious root formation (Table 1, Figs. 1-2). The biomass reduction by WL was more remarkable in *S. jambos* seedlings than in *C. operculatus* seedlings. WL reduced the plant height (PHI) by 60%, the number of blades (BNI) by 50%, the leaf area (LAI) by 36.9%, and the fresh shoot weight (FSW) by 51% in *S. jambos*. Meanwhile, the decrease in biomass in *C. operculatus* was less pronounced (30.25, 47, 34, and 50.6%, respectively) (Table 1). Under stressed and non-stressed conditions, *C. operculatus* displayed stronger morphological development and biomass accumulation (Fig. 1) than *S. jambos* (Fig. 2). The adventitious root formation was induced significantly by WL compared with that in the control in both species. Under submergence conditions, exogenous application of IAA (WL-S-IAA; waterlogging and IAA sprayed, WL-W-IAA; waterlogging and IAA watered) increased the shoot biomass in *C. operculatus* and *S. jambos*, whereas exogenous ABA (WL-S-ABA; waterlogging and ABA sprayed, WL-W-ABA; waterlogging and ABA watered) strongly improved the adventitious root formation and slightly promoted the primary root development. Based on the type of application of exogenous phytohormones, identifying the best type of application that has contributed the most in the development of plant biomass in both species is very complex. Under waterlogging stress, the WL + W-IAA and WL + W-ABA treatments showed the highest values for the biomass parameters (in *C. operculatus*: BNI, fresh shoot weight (FSW) and fresh stem weight (FStW) with WL + W-IAA, adventitious root dry weight (ARsDW), adventitious root fresh weight (ARsFW), primary root dry weight (PRsDW), and primary root fresh weight (PRsFW) with WL + W-ABA; in *S. jambos*: PHI, BNI, LAI, FStW, and PRsFW with WL + W-IAA).

**Table 1:** The plant growth parameters of *C. operculatus* and *S. jambos* under LT-WL and subjected to exogenous ABA and IAA

Species	Treatments	PHI (cm)	BNI	LAI (cm^2^)	FSW (g)	FStW (g)	DStW (g)	ARsFW (g)	ARsDW (g)	PRsFW (g)	PRsDW (g)
*C. operculatus*	**CK**	35.7 ± 2.2 ^a^	38.5 ± 2.5 ^a^	867.9 ± 59.0 ^a^	31.2 ± 5.7 ^a^	33.9 ± 1.2 ^a^	8.2 ± 1.3 ^b^	–	–	66.4 ± 1.8 ^a^	8.9 ± 1.2 ^a^
	**WL**	24.9 ± 3.2 ^c^	20.4 ± 2.8 ^c^	573.1 ± 43.8 ^c^	15.4 ± 1.0 ^c^	27.5 ± 4.1 ^c^	7.8 ± 1.4 ^b^	30,1 ± 6.0 ^b^	4,3 ± 0.60 ^a^	13.4 ± 3.7 ^d^	2.8 ± 0.6 ^c^
	**WL + S-IAA**	30.8 ± 2.7 ^b^	22.8 ± 4.5 ^bc^	788.4 ± 46.9 ^a^	18.4 ± 2.2 ^bc^	31.3 ± 4.0 ^abc^	12.3 ± 1.5 ^a^	33,2 ± 9.1^b^	4,8 ± 1.00 ^a^	16.1 ± 4.7 ^cd^	3.1 ± 1 ^c^
	**WL + W-IAA**	26.2 ± 2.9 ^c^	26.6 ± 2.8 ^b^	671.0 ± 26.9 ^b^	20.6 ± 2.6 ^b^	32.7 ± 2.8 ^ab^	12.0 ± 1.3 ^a^	34,6 ± 3.3 ^ab^	5,4 ± 1.90 ^a^	20.1 ± 5.1 ^bc^	3.4 ± 0.7 ^bc^
	**WL + S-ABA**	24.3 ± 2.9 ^c^	21.6 ± 3.9 ^bc^	502.2 ± 40.2 ^c^	15.8 ± 1.9 ^c^	29.1 ± 1.0 ^bc^	11.1 ± 1.9 ^a^	36,6 ± 2.7 ^ab^	5,4 ± 0.40 ^a^	21.3 ± 2.4 ^b^	4.0 ± 1.0 ^bc^
	**WL + W-ABA**	26.3 ± 3.5 ^c^	19.4 ± 5.9 ^c^	577.9 ± 13.4 ^c^	17.9 ± 3.5 ^bc^	31.6 ± 4.4 ^abc^	11.7 ± 1.4 ^a^	41,8 ± 4.0 ^a^	5,7 ± 0.70 ^a^	23.4 ± 2.3 ^b^	4.6 ± 0.7 ^b^
*S. jambos*	**CK**	26.3 ± 0.9 ^A^	29.0 ± 3.3 ^A^	536.2 ± 94.6 ^A^	19.0 ± 4.7 ^A^	8.0 ± 2.3 ^A^	2.8 ± 0.8 ^A^	–	–	7.6 ± 1.3 ^A^	2.0 ± 0.5 ^A^
	**WL**	10.6 ± 0.9 ^CD^	14.5 ± 2.0 ^CD^	338.5 ± 91.9 ^BC^	9.3 ± 1.1 ^B^	3.0 ± 0.5 ^B^	0.9 ± 0.3 ^C^	1,9 ± 0.6 ^B^	0,3 ± 0.08 ^B^	1.9 ± 0.5 ^B^	0.5 ± 0.1 ^B^
	**WL + S-IAA**	12.6 ± 1.0 ^BC^	15.4 ± 1.9 ^C^	330.4 ± 73.8 ^BC^	10.3 ± 2.0 ^B^	4.5 ± 0.9 ^B^	2.1 ± 0.7 ^B^	2,1 ± 0.4 ^B^	0,4 ± 0.0 ^B^	2.0 ± 0.4 ^B^	0.5 ± 0.2 ^B^
	**WL + W-IAA**	13.2 ± 2.5 ^B^	19.0 ± 3 ^B^	407.0 ± 66.7 ^B^	9.5 ± 1.0 ^B^	4.4 ± 0.7 ^B^	2.1 ± 0.3 ^B^	3,0 ± 0.2 ^A^	0,6 ± 0.09 ^A^	2.8 ± 0.9 ^B^	0.6 ± 0.9 ^B^
	**WL + S-ABA**	11.5 ± 1.0 ^BCD^	13.4 ± 2.7 ^CD^	280.3 ± 49.9 ^C^	9.1 ± 1.5 ^B^	3.6 ± 1.4 ^B^	2.0 ± 0.3 ^B^	3,0 ± 0.1 ^A^	0,7 ± 0.10 ^A^	2.2 ± 0.2 ^B^	0.5 ± 0.1 ^B^
	**WL + W-ABA**	10.0 ± 1.6 ^D^	11.3 ± 2.9 ^D^	332.6 ± 34.4 ^BC^	9.0 ± 1.4 ^B^	3.8 ± 0.6 ^B^	2.0 ± 0.3 ^B^	3,0 ± 0.3 ^A^	0,6 ± 0.06 ^A^	2.7 ± 0.7 ^B^	0.8 ± 0.2 ^B^

Values are expressed as mean ± SE (*n =* 5). The different letters indicate significant differences between treatments (*P <* 0.05) based on Tukey’s multiple comparison test, two way ANOVA. Different lowercases and uppercases indicate significant differences among different treatment. PHI, plant height Increments in; BNI, blades number increment; LAI, leaf area increment; FSW, fresh shoot weight; FStW, fresh stem weight, DStW, dry stem weight; ARsFW, adventitious root fresh weight; ARsDW, adventitious root dry weight; PRsFW, primary root fresh weight; PRsDW, primary root dry weight. ARs roots were not found in CK group.

### Photosynthetic pigment contents and gas exchange evaluation.

The highest photosynthesis activity was found in the non-waterlogged seedlings. The total chlorophyll (T-Chlo) level and net photosynthetic rate (Pn) in both species significantly decreased at the end of the treatment (Table 2). Compared with control plants, *C. operculatus* and *S. jambos* showed decreases of 64.06 and 28.5% of T-Chlo, 48 and 52.63% of Pn, and 30 and 36.36% of transpiration rate (Trr), respectively, under waterlogging stress. The application of exogenous plant hormones in *C. operculatus* seedlings significantly increased the T-Chlo content. According to the type of application, the WL + W-ABA treatment displayed the highest photosynthetic values than the other treatments under submergence conditions. The T-Chlo was increased by 13.2% and the Pn by 3.7% in WL + W-ABA treated plants compared with that in WL-treated seedlings. In *S. jambos* seedlings, WL + S-ABA led to significantly higher Pn than the other treatments under waterlogging conditions. Trr showed better improvement in *S. jambos* seedlings treated with WL + W-ABA. Almost all the photosynthetic parameters were improved by WL + W-ABA in *C. operculatus* under waterlogging but not in *S. jambos* (Table 2).

**Table 2:** Photosynthetic pigments and gas exchanges variations of *C. operculatus* and *S. jambos* under LT-WL and subjected to exogenous IAA and ABA

Species	Treatments	Chlo-*a* (μg/g.Fw)	Chlo-*b* (μg/g.Fw)	Caro (μg/g.Fw)	T-Chlo (μg/g.Fw)	Pn (μmol·m^− 2^·s^− 1^)	Trr (mmol·m^− 2^·s^− 1^)	Gs (mol·m^− 2^·s^− 1^)	Wue (μmol.mmol-1)	Ci (μmol·mol^− 1^)
*C. operculatus*	**CK**	380.2 ± 21.1 ^a^	104.0 ± 21.4 ^a^	245.1 ± 48.8 ^c^	484.2 ± 29.6 ^a^	5.0 ± 0.50 ^a^	2.0 ± 0.3 ^a^	0.080 ± 0.007 ^a^	2.4 ± 0.19 ^a^	441.0 ± 19.7 ^c^
	**WL**	140.4 ± 24.7 ^e^	33.6 ± 5.0 ^d^	328.2 ± 29.1 ^a^	174.0 ± 22.9 ^e^	2.6 ± 0.34 ^c^	1.4 ± 0.2 ^ab^	0.040 ± 0.012 ^cd^	1.8 ± 0.31 ^b^	518.5 ± 25.8 ^a^
	**WL + S-IAA**	211.5 ± 5.43 ^bc^	60.2 ± 16.8 ^bc^	274.5 ± 22.9 ^bc^	271.8 ± 21.1 ^c^	2.8 ± 0.60 ^c^	1.7 ± 0.5 ^ab^	0.048 ± 0.008 ^c^	1.7 ± 0.54 ^b^	516.8 ± 10.5 ^a^
	**WL + W-IAA**	196.2 ± 16.4 ^c^	66.4 ± 17.2 ^b^	309.0 ± 34.3 ^ab^	262.7 ± 14.4 ^c^	2.8 ± 0.30 ^c^	1.7 ± 0.2 ^ab^	0.030 ± 0.006 ^d^	1.7 ± 0.33 ^b^	470.6 ± 51.0 ^bc^
	**WL + S-ABA**	166.8 ± 16.3 ^d^	47.0 ± 7.1 ^cd^	305.5 ± 33.6 ^ab^	213.8 ± 19.6 ^d^	2.6 ± 0.31 ^c^	1.4 ± 0.4 ^b^	0.030 ± 0.013 ^d^	1.9 ± 0.37 ^ab^	465.0 ± 22.1 ^bc^
	**WL + W-ABA**	234.3 ± 16.1 ^b^	70.3 ± 6.5 ^b^	319.5 ± 10.9 ^ab^	304.5 ± 23.3 ^b^	3.5 ± 0.07 ^b^	2.0 ± 0.6 ^a^	0.066 ± 0.013 ^c^	1.8 ± 0.71 ^ab^	492.7 ± 39.7 ^ab^
*S. jambos*	**CK**	375.5 ± 22.9 ^A^	106.3 ± 14.9 ^A^	98.5 ± 28.1 ^A^	481.8 ± 24.7 ^A^	5.7 ± 0.6 ^A^	2.2 ± 0.2 ^A^	0.062 ± 0.013 ^A^	2.6 ± 0.29 ^A^	451.2 ± 17.7 ^BC^
	**WL**	278.3 ± 36.1 ^CD^	66.2 ± 8.1 ^BC^	85.3 ± 12.8 ^AB^	344.5 ± 42.3 ^C^	2.7 ± 0.5 ^BC^	1.4 ± 0.2 ^B^	0.036 ± 0.008 ^B^	1.8 ± 0.18 ^B^	496.8 ± 14.6 ^A^
	**WL + S-IAA**	283.4 ± 35.7 ^CD^	71.9 ± 10.2 ^B^	68.9 ± 8.7 ^B^	355.4 ± 37.7 ^BC^	2.4 ± 0.3 ^C^	2.2 ± 0.4 ^A^	0.056 ± 0.016 ^A^	1.6 ± 0.34 ^BC^	420.2 ± 23.8 ^C^
	**WL + W-IAA**	315.7 ± 16.7 ^B^	72.8 ± 11.7 ^B^	77.5 ± 11.6 ^AB^	388.5 ± 20.1 ^B^	2.6 ± 0.4 ^BC^	2.0 ± 0.3 ^A^	0.050 ± 0.012 ^AB^	1.3 ± 0.42 ^CD^	480.6 ± 28.2 ^AB^
	**WL + S-ABA**	265.7 ± 19.9 ^D^	56.6 ± 9.8 ^C^	67.5 ± 6.6 ^B^	322.4 ± 28.8 ^C^	3.3 ± 0.3 ^B^	2.2 ± 0.5 ^A^	0.052 ± 0.004 ^B^	1.1 ± 0.12 ^D^	426.7 ± 32.3 ^C^
	**WL + W-ABA**	309.6 ± 7.5 ^BC^	80.3 ± 7.2 ^B^	81.0 ± 17.6 ^AB^	390.0 ± 11.9 ^B^	2.8 ± 0.2 ^C^	2.2 ± 0.2 ^A^	0.050 ± 0.010 ^AB^	1.2 ± 0.16 ^CD^	473.7 ± 19.6 ^AB^

Values are expressed as mean ± SE (*n =* 5). The different letters indicate significant differences between treatments (*P <* 0.05) based on Tukey’s multiple comparison test, two way ANOVA. Different lowercases and uppercases indicate significant differences among different treatments. Chlo-*a*; chlorophyll a, Chlo-*b*; chlorophyll b, Caro; carotenoid Gs; stomatal conductance, Wue; water use efficiency, Ci; intercellular CO_2_ concentration.

### Relative conductivity (RC), superoxide anions (O_2_^.–^) production, and malondialdehyde (MDA) content in leaves

**Fig. 3:**
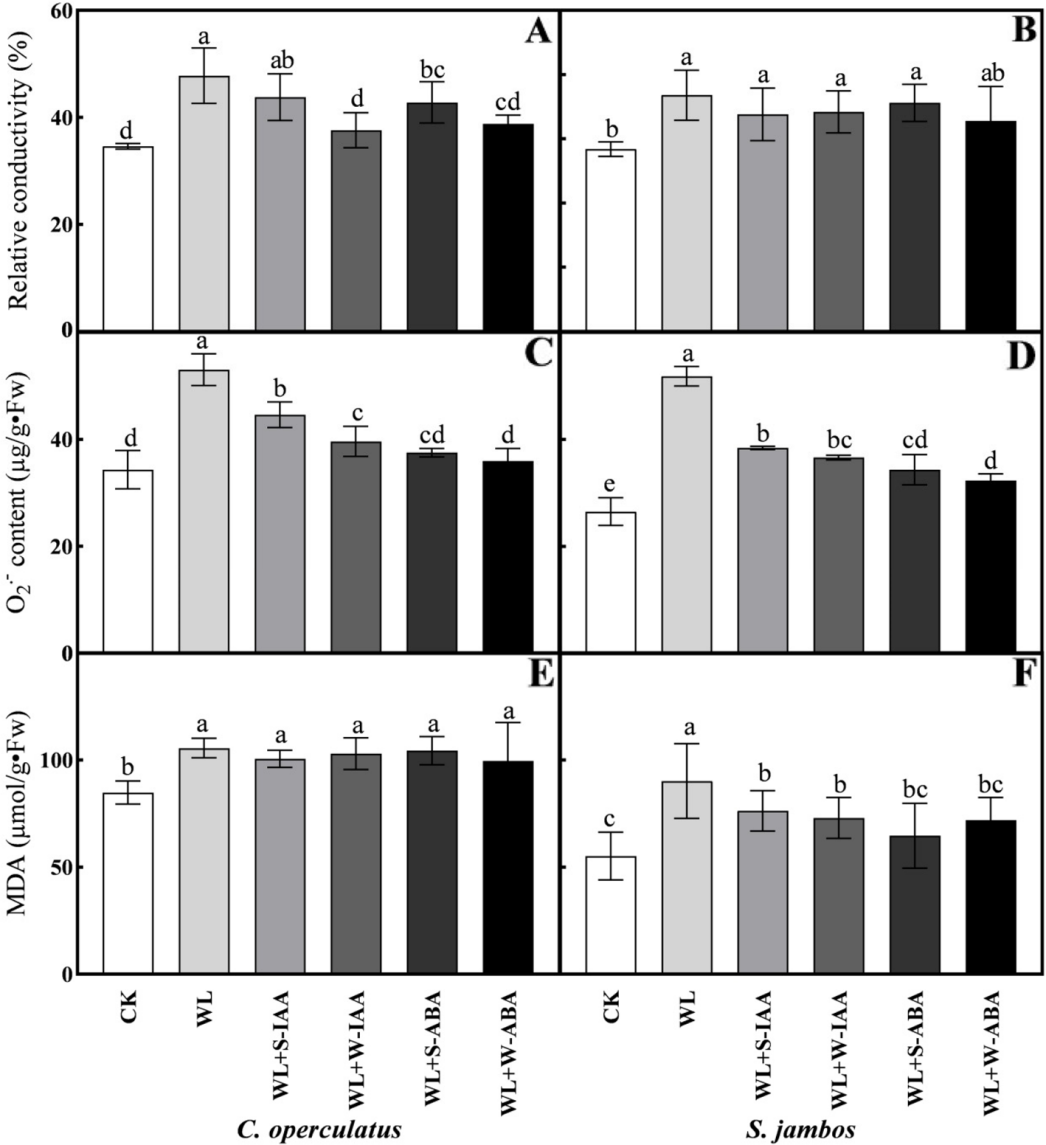
Relative conductivity RC (**A**-**B**), O_2_^.–^ accumulation (**C**-**D**), and MDA content (**E**-**F**) of *C. operculatus* and *S. jambos*, respectively, under LT-WL and subjected to exogenous plant hormones. The bars on the top show SE (*n =* 5), and different letters indicate significant differences according to Tukey’s multiple comparison test (*P <* 0.05)

The leaf RC for both species under waterlogged condition was significantly higher than that in the control plants (Fig. 3A-B). The RC content decreased more in *C. operculatus* by exogenous phytohormone application than *S. jambos* under submergence conditions. *S. jambos* seedlings that were waterlogged and subjected to exogenous phytohormones had statistically similar RC than those in WL-treated seedlings (Fig. 3B). The WL + W-IAA treatment led to lower RC the other treatments in waterlogged *C. operculatus* seedlings. In both species, the O_2_^.–^ production was significantly higher under waterlogging stress, while exogenous phytohormones remarkably decreased the O_2_^.–^ content under submergence conditions (Fig. 3C-D). The O_2_^.–^ content in both species was lower in the WL + W-ABA treatment than in the other treatments. The MDA contents were statistically same in *C. operculatus* seedlings under waterlogged conditions and subjected to exogenous phytohormones. Meanwhile, in *S. jambos* plants, exogenous phytohormones reduced drastically the MDA accumulation under submergence conditions (Fig. 3E-F). Low MDA contents in *S. jambos* were found in the WL + S-ABA treatment under waterlogging stress (Fig. 3E).

### Soluble protein and proline contents

**Fig. 4:**
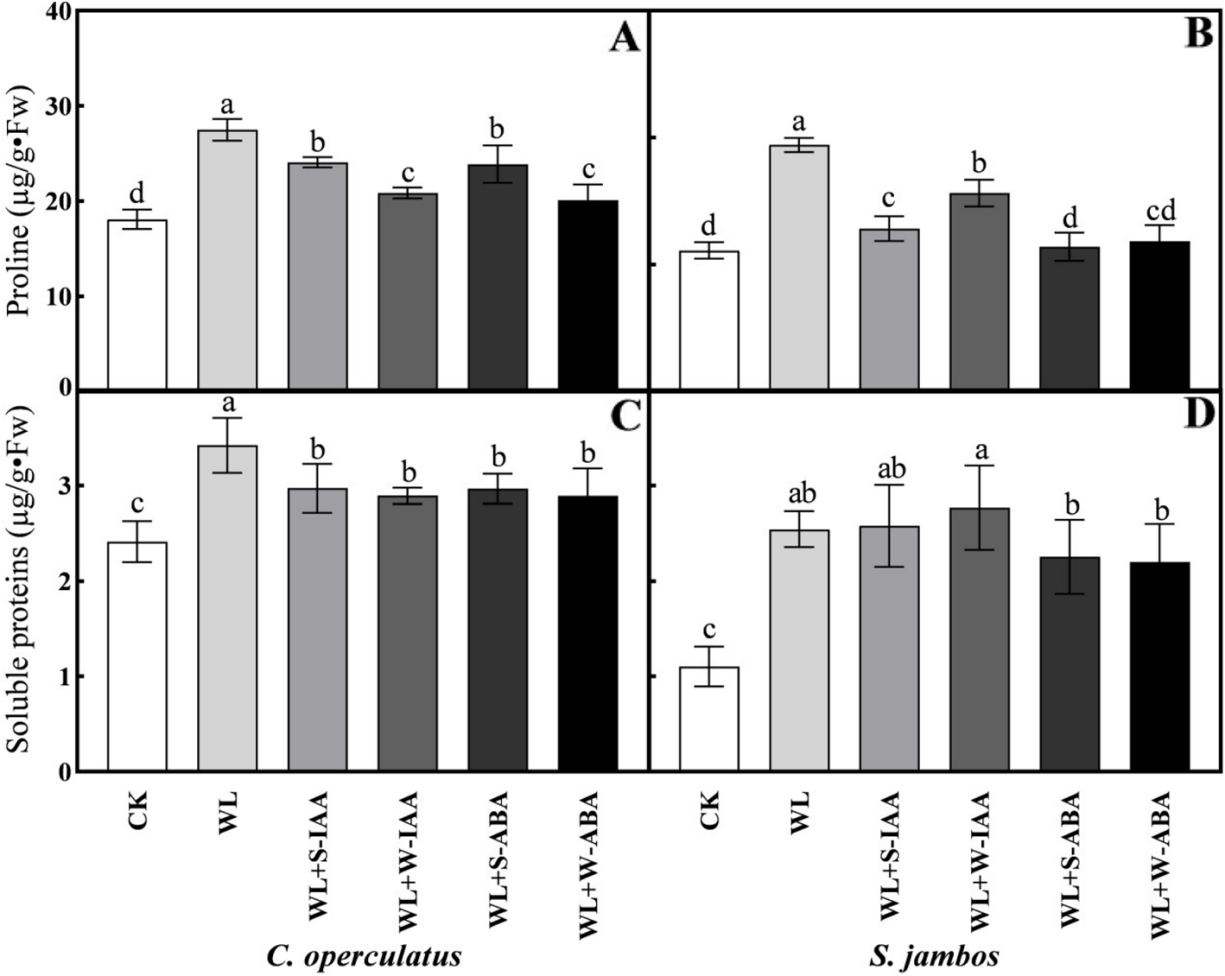
Proline content (**A-B**) and soluble protein concentration (**C-D**) of *C. operculatus* and *S. jambos* under LT-WL and subjected to exogenous plant hormones. The bars on the top show SE (*n =* 5), and different letters indicate significant differences according to Tukey’s multiple comparison test (*P <* 0.05)

The levels of soluble protein and proline in the leaves of *C. operculatus* and *S. jambos* under waterlogged conditions were higher (*p* ≤ 0.05) than those in the control (Fig. 4). In waterlogged pots without exogenous phytohormones, the proline content increased strongly compared with that in the samples subjected to ABA or IAA for both species. The concentration of protein in WL-treated seedlings was significantly increased in *C. operculatus*, while the protein content was statistically similar between WL-treated and IAA-treated seedlings (Fig. 4C).

### Antioxidant systems

**Fig. 5:**
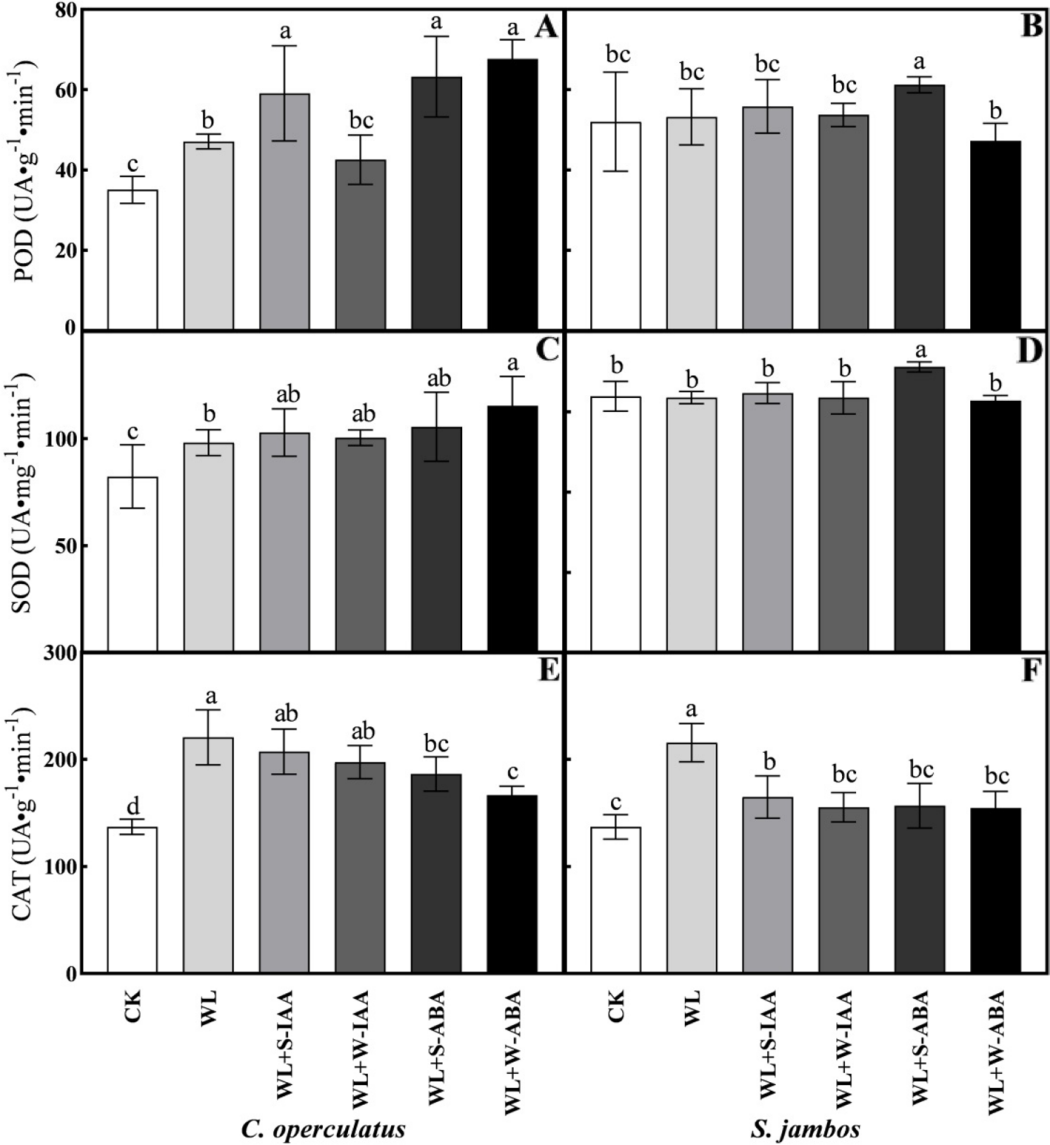
The activities of POD (**A**-**B**), SOD (**C**-**D**), and CAT (**E**-**F**) of *C. operculatus* and *S. jambos* under LT-WL and subjected to exogenous plant hormones. The bars on the top show SE (*n =* 5), and different letters indicate significant differences according to Tukey’s multiple comparison test (*P <* 0.05)

**Fig. 6:**
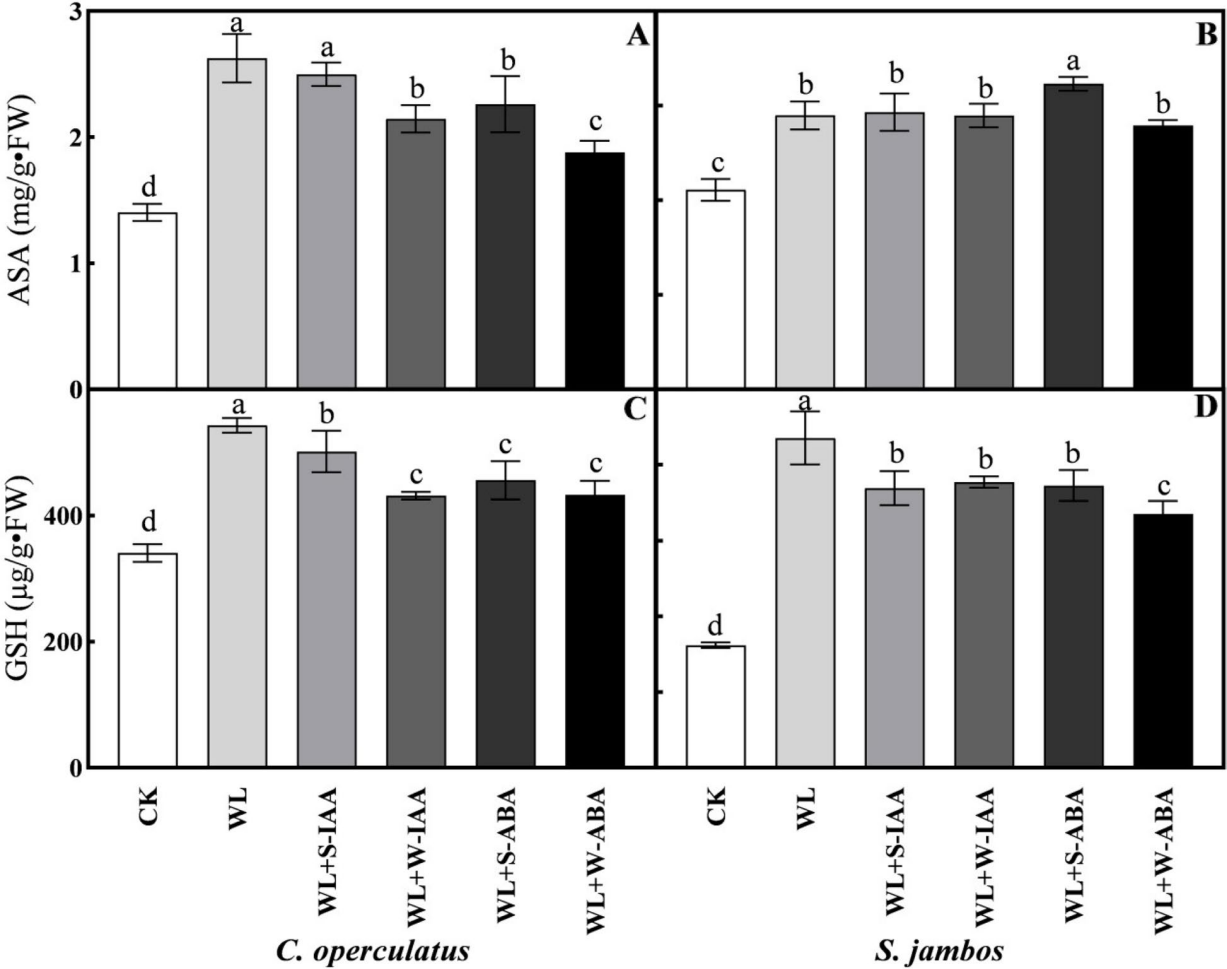
The contents of ASA (**A**-**B**) and GSH (**C**-**D**) of *C. operculatus* and *S. jambos* under LT-WL and subjected to exogenous plant hormones. The bars on the top show SE (*n* = 5), and different letters indicate significant differences according to Tukey’s multiple comparison test (*P <* 0.05)

The WL treatment significantly increased the antioxidant capacity of *C. operculatus* in comparison with that under normal conditions, as reflected by the following: increases of 34.35% in the POD activity, 19.13% in the SOD activity, and 60.95% in the CAT activity in WL-treated seedlings compared with the control (Fig. 5). The POD activity in *C. operculatus* seedlings under stress conditions was statistically similar among WL + W-ABA, WL + S-ABA, and WL + S-IAA treatments (Fig. 5A). Meanwhile, the activity was significantly higher in WL + W-ABA seedlings than in the other treatments. Most of the exogenous phytohormones treatments did not increase the SOD activities in both species under stress conditions compared with WL treatment. However, the SOD activity was increase in *C. operculatus* by WL + W-ABA and in *S. jambos* by WL + S-ABA. Moreover, the CAT activity was not increase by exogenous phytohormones in both species. The levels of ascorbic acid (ASA) and glutathione reduced (GSH) accumulated in *C. operculatus* were higher in WL-treated seedlings than those in the control, regardless of the exogenous phytohormones applied (Fig. 6). These changes were not statistically significant between WL-treated seedlings and seedlings exposed to WL + S-IAA under stress. *S. jambos* seedlings showed higher accumulation of ASA and GSH in WL-treated seedlings than in the other treatments.

### Plant hormone contents

In the current study, waterlogging was associated with significant changes in plant hormone profiles depending on the type of treatment or plant organ used at all levels (Table 3). The levels of ABA, IAA, JA-Me, and gibberellic acid (GA_3_) in *C. operculatus* and *S. jambos* leaves showed very significant decreases at the end of the experiment due to soil waterlogged conditions compared with those in the control (Table 3). The ABA contents in *C. operculatus* ARs were reduced by WL treatment in comparison with other treatments; WL + W-ABA treatment increased the ABA levels by 60.8% compared with WL treatment. In *S. jambos* ARs, WL + S-IAA increased the ABA content by 20.9% compared with WL treatment (Table 3). Under waterlogging conditions in *C. operculatus*, WL + W-ABA and WL + S-IAA enhanced the IAA levels in ARs by 59.7 and 9.6%, respectively, compared with WL treatment. Under WL treatment, WL + W-ABA and WL + S-ABA treatments significantly decreased the ABA and GA_3_ contents in ARs of *S. jambos* compared with WL treatment. The GA_3_ in Ars of *C. operculatus* was strongly increased by WL + W-ABA, but it decreased significantly in *S. jambos*. Under submergence conditions, higher GA_3_ content in ARs was found in WL + W-ABA-treated seedlings in *C. operculatus* (Table 3). In *S. jambos*, WL- and WL + S-IAA-treated seedlings displayed greater values. Furthermore, in the ARs of *C. operculatus* seedlings, the accumulation of JA-Me was significantly higher after WL + S-IAA, WL + W-IAA, WL + S-ABA, and WL + W-ABA treatments than that after WL treatment. In contrast to the ARs of *S. jambos*, only WL + S-IAA increased the JA-Me content. Although the JA-Me contents in leaf were drastically reduced by WL treatment associated or not with exogenous phytohormones (ABA or IAA).

**Table 3:** *C. operculatus* and *S. jambos*’ ABA, IAA, GA_3_, and JA-Me contents in Ars and leaves under LT-WL and subjected to exogenous plant hormones

Species	Treatments	ABA in ARs	IAA in ARs	GA_3_ in ARs	JA-Me in ARs	ABA in leaf	IAA in leaf	GA_3_ in leaf	JA-Me in leaf
*C. operculatus*	**CK**	**–**	**–**	**–**	**–**	142.5 ± 6.2 ^a^	37.5 ± 2.3 ^a^	6.7 ± 0.1 ^a^	29.2 ± 1.5 ^a^
	**WL**	51.2 ± 2.2 ^c^	29.8 ± 1.7 ^c^	3.7 ± 0.1 ^b^	8.2 ± 0.2 ^d^	128.8 ± 6.4 ^b^	21.9 ± 0.7 ^c^	5.4 ± 0.1 ^c^	21.5 ± 1.7 ^b^
	**WL + S-IAA**	59.2 ± 3.8 ^b^	32.7 ± 1.4 ^b^	3.4 ± 0.2 ^c^	13.1 ± 0.3 ^c^	103.2 ± 6.5 ^d^	21.8 ± 1.6 ^c^	5.4 ± 0.1 ^c^	17.5 ± 0.9 ^c^
	**WL + W-IAA**	52.2 ± 2.5 ^c^	23.7 ± 1.0 ^e^	3.4 ± 0.1 ^c^	15.8 ± 0.4 ^b^	127.9 ± 3.5 ^b^	28.4 ± 1.5 ^b^	5.4 ± 0.3 ^c^	9.8 ± 0.5 ^d^
	**WL + S-ABA**	62.0 ± 4.2 ^b^	26.5 ± 1.1 ^d^	3.6 ± 0.1 ^bc^	16.0 ± 0.4 ^b^	114.2 ± 6.0 ^c^	36.8 ± 2.7 ^a^	5.8 ± 0.1 ^b^	16.7 ± 0.4 ^c^
	**WL + W-ABA**	82.3 ± 2.0 ^a^	47.7 ± 1.8 ^a^	4.4 ± 0.2 ^a^	22.8 ± 0.5 ^a^	117.6 ± 6.1 ^c^	21.6 ± 1.5 ^c^	4.8 ± 0.2 ^d^	20.0 ± 1.1 ^b^
*S. jambos*	**CK**	**–**	**–**	**–**	**–**	109.9 ± 4.1 ^A^	32.0 ± 1.3 ^A^	6.4 ± 0.3 ^A^	22.8 ± 1.5 ^A^
	**WL**	23.2 ± 1.4 ^B^	15.9 ± 0.7 ^B^	3.7 ± 0.1 ^A^	14.0 ± 0.8 ^BC^	74.2 ± 3.0 ^BC^	12.3 ± 0.5 ^D^	2.4 ± 0.1 ^D^	11.4 ± 0.4 ^B^
	**WL + S-IAA**	29.3 ± 2.2 ^A^	17.1 ± 0.5 ^AB^	3.6 ± 0.1 ^A^	19.3 ± 0.7 ^A^	61.9 ± 1.6 ^D^	15.9 ± 0.4 ^B^	3.7 ± 0.1 ^B^	10.1 ± 0.6 ^C^
	**WL + W-IAA**	23.9 ± 1.7 ^B^	17.0 ± 1.0 ^AB^	2.8 ± 0.0 ^B^	13.9 ± 1.0 ^C^	58.7 ± 2.2 ^D^	13.4 ± 0.8 ^C^	3.8 ± 0.1 ^C^	9.6 ± 0.5 ^CD^
	**WL + S-ABA**	20.7 ± 2.2 ^C^	15.8 ± 0.5 ^B^	2.6 ± 0.1 ^C^	13.0 ± 0.5 ^C^	77.7 ± 1.7 ^B^	16.2 ± 0.6 ^B^	3.3 ± 0.2 ^C^	8.6 ± 0.2 ^D^
	**WL + W-ABA**	16.3 ± 1.2 ^D^	16.7 ± 0.4 ^B^	2.7 ± 0.1 ^C^	15.0 ± 0.7 ^B^	70.5 ± 2.0 ^C^	13.8 ± 0.7 ^C^	3,6 ± 0.1 ^B^	10.0 ± 0.4 ^C^

Values are expressed as mean ± SE (*n =* 6). The different letters indicate significant differences between treatments (*P <* 0.05) based on Tukey’s multiple comparison test, two way ANOVA. Different lowercases and uppercases indicate significant differences among different treatments. The phytohormones in ARs roots were not found in CK group.

### Phytohormonal and physiological data network analysis and correlation

**Fig. 7:**
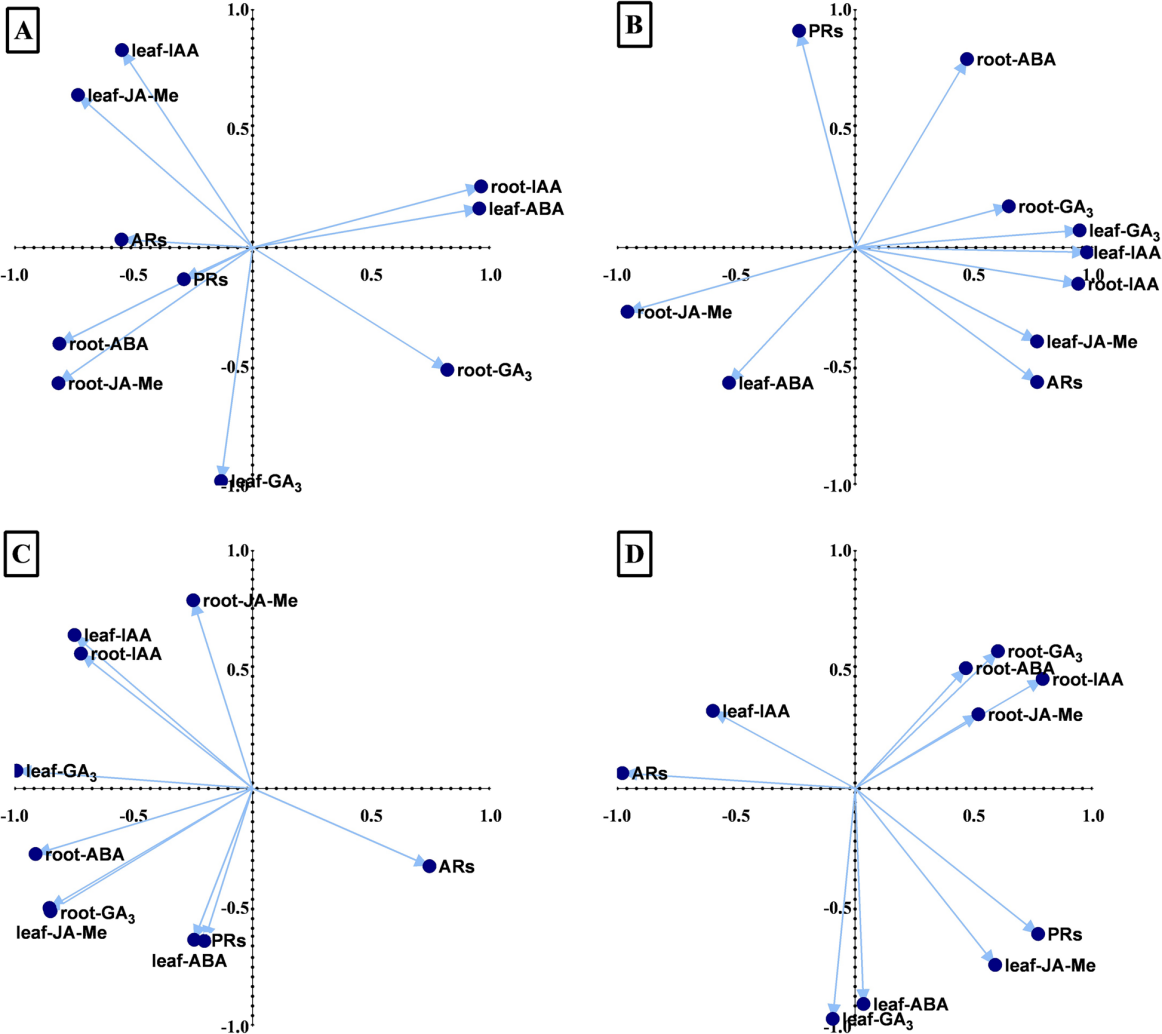
Plot of the first two PCAs showing correlation between the phytohormones, ARs and PRs biomass accumulation under LT-WL and subjected to exogenous IAA (**A**-**B**) and ABA (**C**-**D**) in *C. operculatus*

**Fig. 8:**
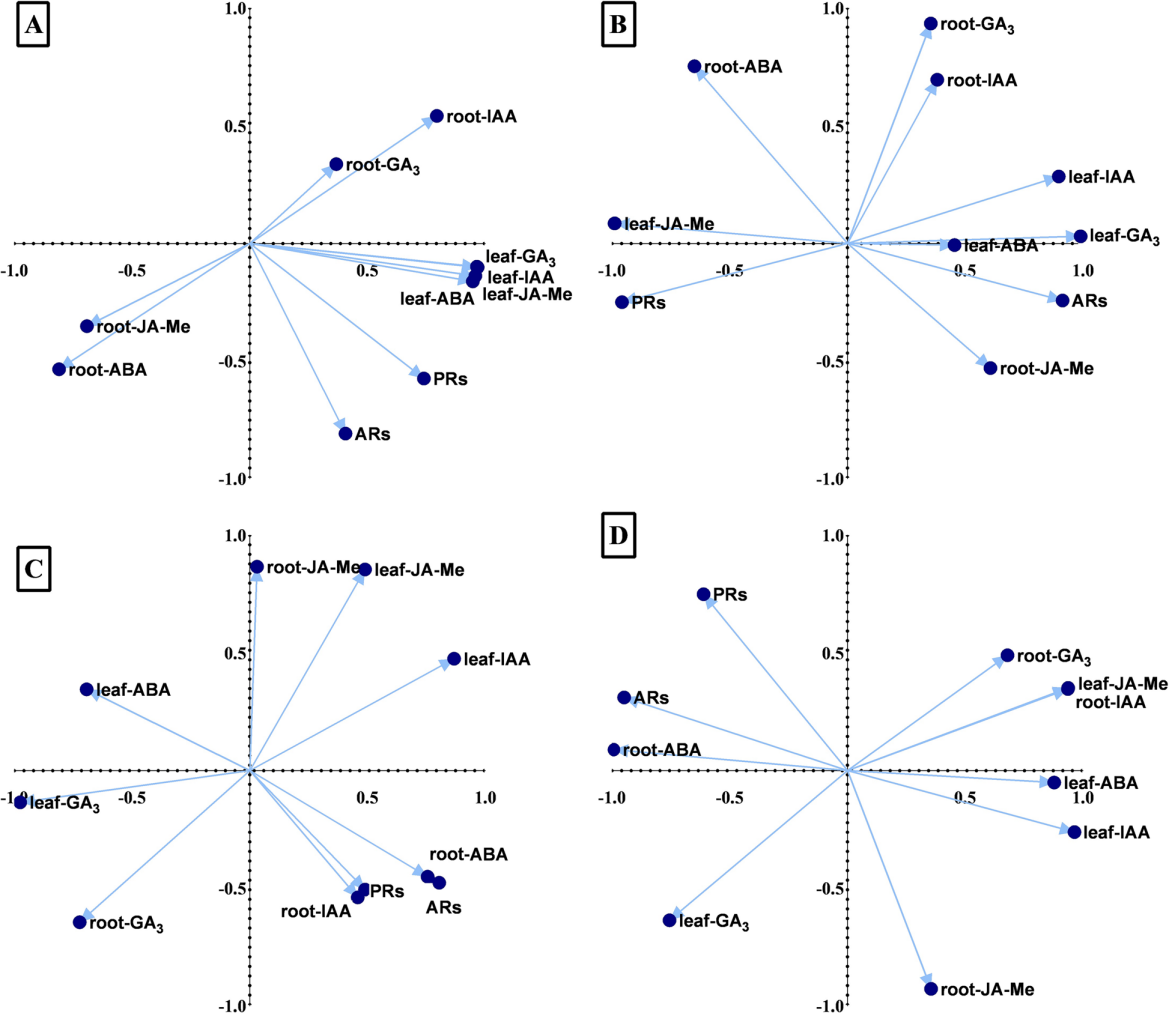
Plot of the first two PCAs showing correlation between the phytohormones, ARs and PRs biomass accumulation under LT-WL and subjected to exogenous IAA (**A**-**B**) and ABA (**C**-**D**) in *S. jambos*

Principal component analysis (PCA) of loading data was performed to determine the correlation between PRs, AR formation, and plant hormones under different treatments (Figs. 7-8). The results revealed no significant similarities in the association profile of the selected parameters (plant hormones, PRs, and ARs) according to the type of species or treatment. These dissimilarities were found in the comparison among PCA bi-plots belonging to *C. operculatus* (Figs. 7A-D) or *S. jambos* (Figs. 8A-D) under different treatments and stress condition. The same phenomenon was observed with the comparison of PCAs between *C. operculatus* and *S. jambos*. In *C. operculatus* seedlings under WL, a significant association was found between PRs, ARs, AR JA-Me content, and AR ABA contents under WL + S-IAA (Fig. 7A) treatment and based on the first component. A strong association also existed between PRs and leaf endogenous ABA under WL + S-ABA based on PC1 and PC2 (Fig. 7C). Under WL + W-ABA treatment, only endogenous JA-Me showed a positive correlation with PRs. Meanwhile, ARs did not show any strong positive correlation with various plant hormones (Fig. 7D). However, in *S. jambos* seedlings, the ARs displayed greater association with endogenous ARs-ABA content under WL + W-ABA (Fig. 8) compared in *C. operculatus*. Based on PC1 and PC2, a significant association was found among ARs-ABA content, ARs, ARs-IAA content, and PR in *S. jambos* under WL + S-ABA. In *C. operculatus*, a group of physiological indicators including antioxidants, MDA, O_2_^.–^, proline, proteins, RC, and Ci were the most strongly correlated with WL, WL-S-IAA, and WL-W-IAA. Meanwhile, the data from the phytohormones showed association with the control treatment, except for endogenous IAA, GA_3_, and ABA in ARs (Suppl. Fig. 1A). In *S. jambos* seedlings, the following indicators involving photosynthetic data, growth data, and plant hormones besides endogenous JA-Me, GA_3_, and ABA in ARs seemed to be more influenced by the control treatment. WL-S-IAA, WL-W-IAA, WL-S-ABA, and WL-W-ABA showed very significant correlations with the physiological and hormonal data (Suppl. Fig. 1B). Moreover, the Fuzzy membership functions suggested that WL-W-ABA treatment showed the most positive effect on both species under water stress (Suppl. Table 1-2).

## Discussion

**Fig. 9:**
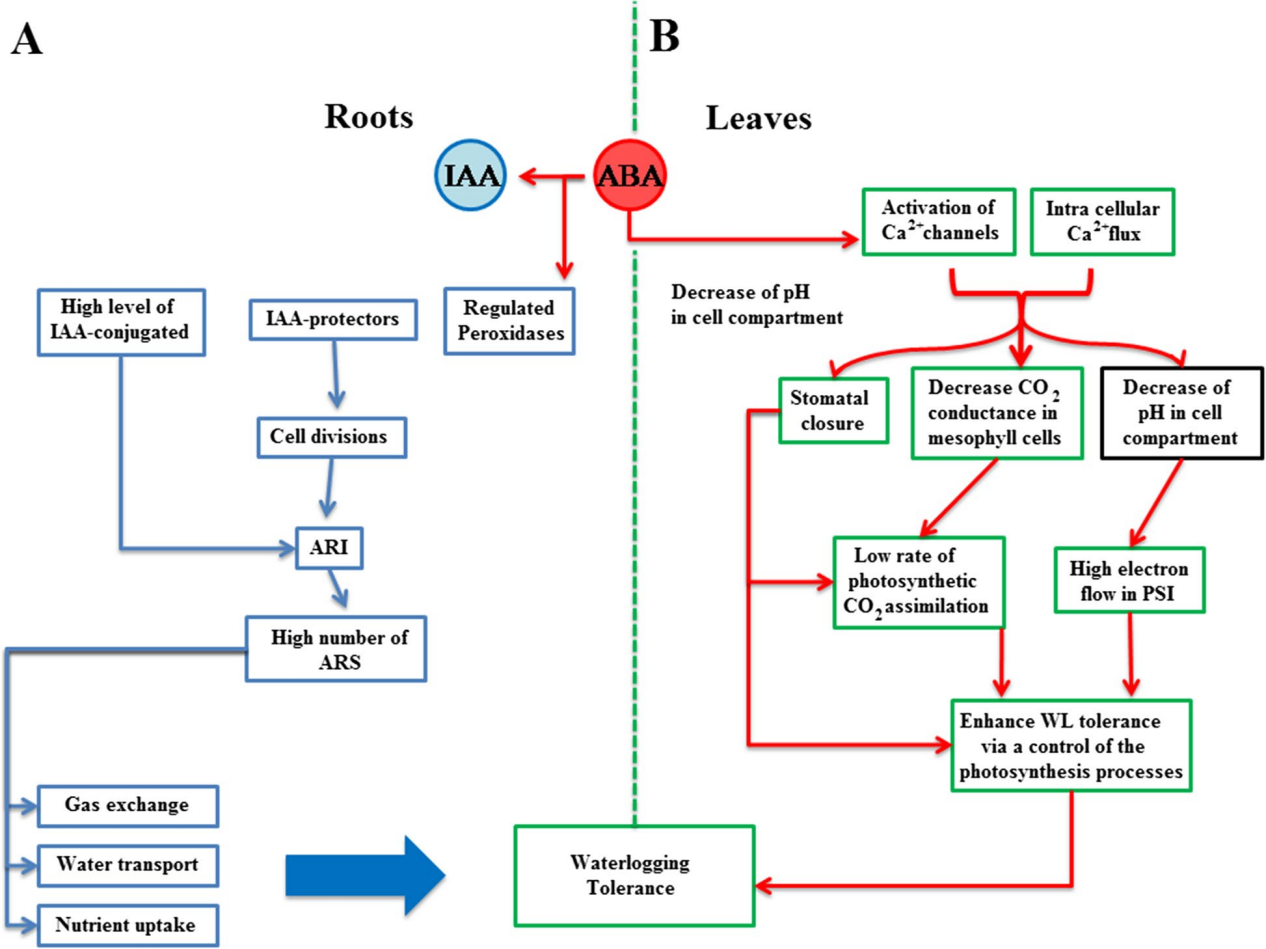
Examples of different pathway which exogenous ABA regulate adventitious root formation (**A**) and photosynthesis (**B**)

Trees have possessed diverse complex mechanisms to help them counter/acclimate to harsh environmental conditions, such as waterlogging or flooding stresses. Waterlogging stress alter the functioning of various fundamental plant processes, particularly photosynthesis [36]. Waterlogging reduces plant growth, photosynthetic pigment activity, Gs, and Trr. Numerous plant hormones, such as IAA or ABA, are deeply involved in the physiological, biochemical, and hormonal adaptive responses of plants under submergence conditions [6]. There is an earlier work [18] that reported that the formation and development of ARs via exogenous ABA provided a glimmer of light of the positive role of ABA in plants under submergence conditions (Fig. 9A).

### Plant growth indices and photosynthesis in both tree species under LT-WL and subjected to exogenous hormones (IAA and ABA)

Plant hormones, such as ABA, have been used exogenously to protect plant photosynthesis under submergence conditions (Fig. 9B, [16]. ABA and IAA were used exogenously to shed light on their roles in woody plant under long-term waterlogging conditions. Our results suggest that *C. operculatus* showed greater waterlogging tolerance due to its ability to possess greater leaves, longer roots, and stem compared with *S. jambos*. The plant’s length, width, or height can influence the physiological and morphological adaptive responses of plants to environmental conditions [37]. Moreover, it has been reported that larger plants are more invulnerable to harsh conditions, which is related to improved water availability, considering that larger plants generally have deeper roots [38]. However, it has been suggested that under warming conditions, large leaves may be particularly detrimental to the photosynthetic performance of plant species [39]. Indeed, it has been proposed a model in which small leaf probably had an advantage against stress related to temperatures. The results of the present work are correlated with those reported in [38]. Thus, the role of plant’s size against abiotic stress depends on the type of stress. Under waterlogging condition, *C. operculatus* displayed a higher internal CO_2_. High internal CO_2_ levels may help in reducing photo-inhibition during stomatal closure. Moreover, the present study showed a decrease in endogenous ABA content in *C. operculatus* seedlings by exogenous ABA under LT-WL. The highest Pn value was found in seedlings treated with ABA under LT-WL. Hence, ABA was moderated and was not harmful for plant growth or photosynthesis of both species under stress conditions. Moreover, a previous study showed that the application of exogenous ABA to the ABA-deficient barley increased the root and root cell hydraulic conductivity [26]. Exogenous ABA acted more in improving the primary root formation that allowed both species to inhibit the reduction of root growth and nodulation caused by the lack of O_2_ induced by submergence conditions [40], thus affecting positively the photosynthesis. Further, based on previous studies, ABA plays a major role in promoting PSII efficiency in plants under salinity or heavy metal stress [41]. In another hand, the seedlings of both species that were subjected to LT-WL treatment associated with auxin showed statistically similar Pn and photosynthetic pigment concentrations in comparison with those exposed to WL-treatment. The results of the present study do not take anything from the role of exogenous IAA in improving photosynthesis in plant under abiotic stresses. Several reports established that exogenous IAA can improve photosynthesis and growth in *W. somnifera* under ultraviolet-B radiation [21] or increased chlorophyll concentration in *Trifoliumrepens repens* under water stress [20]. In *Helianthus annuus* under heavy metal stress, exogenous auxin is able to improve the capability of energy trapping by PSII reaction centers and provides a substantial preservation of the photosynthetic pigments [42]. Leaf photosynthesis and shoot biomass accumulation are pivotal factors for adventitious roots formation [43]. Both plant hormones can improve the shoot and root biomass under LT-WL, particularly exogenous ABA which showed a great ability to increase the ARs formation in both species under water stress. The results suggest that under LT-WL stress, a convenient concentration of ABA or IAA application can release the damage caused by waterlogging stress in plant photosynthesis apparatus that may affected positively the roots and ARs accumulation in both tree species. Indeed, auxin accumulation in the base of the plant can induce growth of pre-formed root initials [44]. Also, it has been reported that ABA may trigger adventitious root initiation in mung bean by regulating IAA [18]. However, WL + S-IAA and WL + W-IAA increased better the above-ground in both species compared WL + S-ABA and WL + W-ABA. WL + W-ABA had showed massive increased of the below-ground under waterlogging in *C. operculatus*.

### Oxidative and membrane damages in *C. operculatus* and *S. jambos* under LT-WL and its mitigation by exogenous IAA and ABA

Waterlogging as hypoxic stress is able to induce the aggregation of a large number of toxic phytohormones in plant organs. Indeed, various plant species diminish their stomatal conductance and photosynthetic activity to avoid water loss and abiotic stress, leading to ROS accumulation [45–47]. The cell’s membrane and function degradation under LT-WL is for the most part induced by excessive ROS accumulation caused by excessive water content that interrupted electrolyte transmission in plant cells [48]. Moreover, ROS accumulation can generate lipid peroxidation protein and DNA degradation [49]. Under environmental stresses, plants use various mechanisms, such as antioxidant enzyme formation or compatible solute accumulation, to counteract inordinate MDA and ROS in the plant cells [50]. The results suggest that the over-accumulation of superoxide anions and MDA induced by LT-WT was relieved by exogenous IAA and ABA. The decrease of O_2_^.–^ content by exogenous plant hormone in both species was accompanied by a decrease of RC in *C. operculatus* and a decline of MDA in *S. jambos*. Proline is one of the most effective osmoprotectant in plants under abiotic stress, the results showed a high accumulation of proline in both species under LT-WT, and the addition of exogenous ABA or IAA did not increase its biosynthesis. In the present study, almost all antioxidant enzymes (POD, SOD, and CAT) or molecules (proline, ASA, and GSH) that have been involved in the regulation or scavenging of ROS and MDA in both species under LT-WL increased strongly. Indeed, it has been well-established that submergence conditions can enhance drastically the activity of the osmoprotectant or anti-oxidant machineries to protect plant cells from membrane damages and oxidative stress. The endogenous ABA and IAA contents in *C. operculatus* and *S. jambos* leaves were lower in LT-WL compared to the control. It seems that lower accumulation of endogenous ABA or IAA in plants under LT-WL reflects the lower antioxidant activity and accretion, in addition to decrease the proline concentration. It has been showed that ABA accumulation can increase the formation of ROS, leading to the induction of the antioxidant defense system in maize leaves [51]. Moreover several studies mentioned the involvement of exogenous IAA in the increase in antioxidant activities in plants under stress [21], [41], [52]. However, mechanisms by which exogenous IAA might increase the antioxidant activity remain unclear. Based on the inconsistency of the results to display one treatment that was able to show significant ROS scavenging, MDA inhibition and antioxidant increment at all levels, it is difficult to suggest which treatment had a better effect to alleviate the oxidative damages. Nonetheless, the spray or watering of exogenous IAA and ABA might probably key roles in releasing oxidative stress under waterlogging conditions.

### Phytohormonal profile and crosstalk with ARs formation in both tree species subjected to exogenous IAA and ABA under LT-WL

**Fig. 10:**
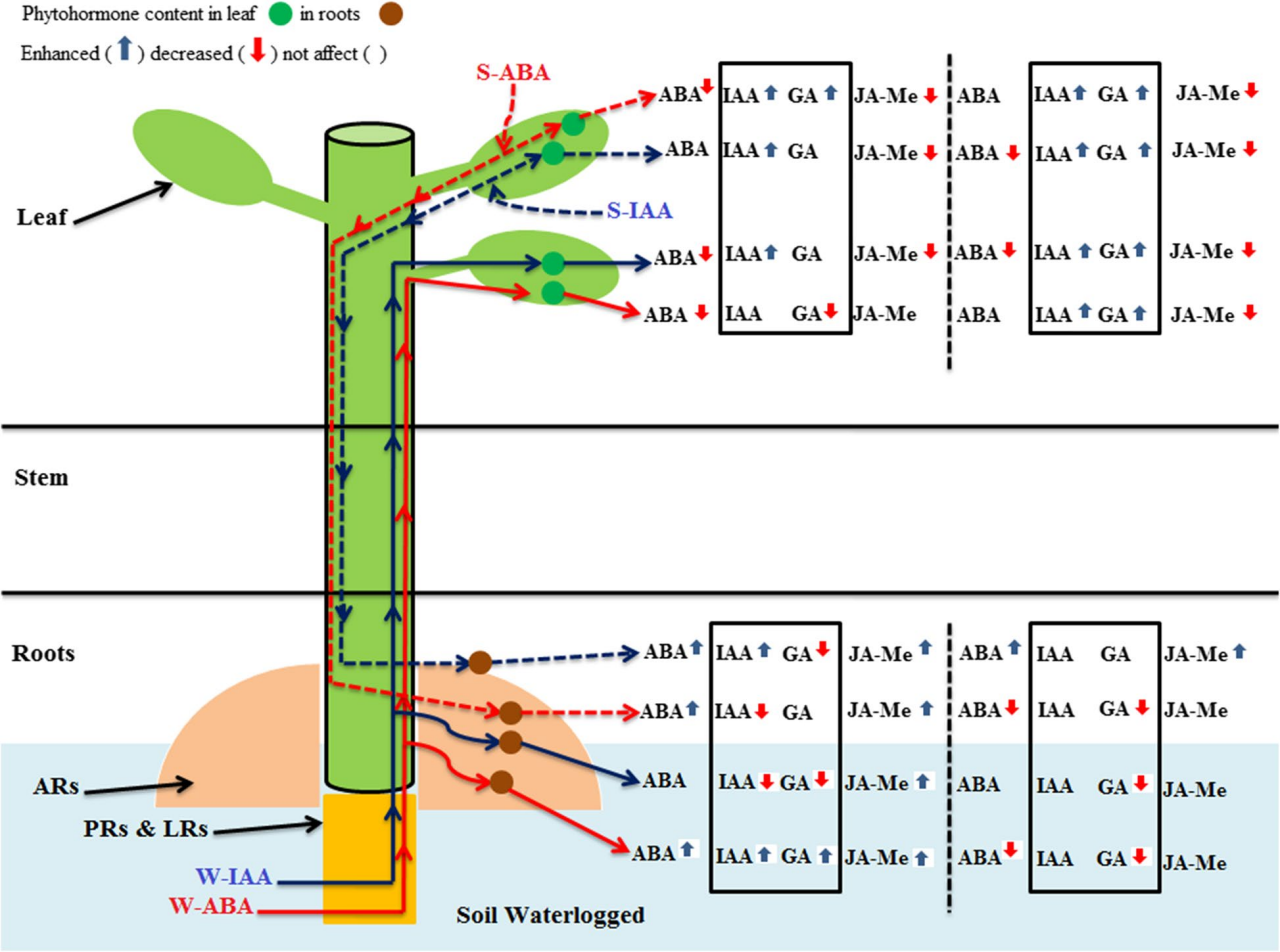
Phytohormonal profiles in the leaves and adventitious roots of *C. operculatus* and *S. jambos* under LT-WL conditions

Waterlogging can restrict axile root elongation and lateral root formation and stimulate adventitious and axile root emergence as well as aerenchyma formation. These processes are deeply modulated by variations in endogenous phytohormones [53]. Plant hormones, such as GAs or auxins, are one of the prominent plant regulators that are involved in controlling the size and number of cells in plant under waterlogging at different stages of ARs formation [6], [54]. The present study showed that endogenous accumulation of IAA in ARs belonging to the stronger tolerant waterlogging species *C. operculatus* was higher under LT-WL than that in *S. jambos* (Fig. 10). However, *S. jambos* (moderate waterlogging-tolerant species) that possesses smaller morphological characteristics than *C. operculatus* did not show a higher IAA accumulation in its ARs under LT-WL.

The result may suggest that the most tolerant plant species that showed higher ARs biomass and development had a greater IAA accumulation in ARs under LT-WL. In both species, GA_3_ contents in ARs were only increased by WL + W-ABA treatment compared to WL treatment. Under submergence conditions, GA is fruitless on its own but acts in a harmonious pathway with ethylene to boost the number of penetrating roots and the growth rate of emerged roots [7], [55]. Meanwhile, it has been well-established that IAA modulates directly adventitious root development by interplaying with ethylene under submergence conditions. In fact, inhibitor studies using N^− 1^-naphthylphthalamic acid showed that polar auxin transport through the PIN-FORMED (PIN) family of auxin efflux carriers is needed for adventitious root growth in rice under flooding [7], [56].

Furthermore, the data suggest that exogenous IAA and ABA promoted the down or up-regulation of IAA and ABA in tree roots and leaves during LT-WL following the degree of waterlogging-tolerance that possess the plant species or the type of application of the exogenous plant hormone during the experiment. The supply of these regulators might affect their own biosynthesis in long term. The application of exogenous of ABA or IAA in a LT-WL may not increase the content or biosynthesis of ABA in both leaf species. The exogenous application of ABA via chemigation seemed to increase endogenous IAA in the leaves of both species. The present study showed the huge benefit effect of a supply of ABA in plant waterlogging tolerance via the promotion of adventitious roots and primary roots. The endogenous ABA accumulation in ARs was more accentuated in stronger waterlogging-tolerant specie (*C. operculatus*) than in moderate waterlogging-tolerant plant (*S. jambos*), which is a very curious circumstance in different facet. Indeed, in short term of submergence conditions it has been well documented the antagonist role of ABA in GA biosynthesis and ethylene action in ARs formation. It was reported that a short term of submergence conditions increased endogenous ABA and waterlogging tolerance [7], [17], [27], [57]. The results showed that a supply of ABA via spraying or chemigation technique under LT-WL did not increase the endogenous ABA in ARs or inhibited the GA_3_ accumulation in *C. operculatus*. However, the antagonist action of ABA was found in *S. jambos* that showed a decrease in GA_3_ under LT-WL and subjected to exogenous ABA. As long as we can provide results that support the role of exogenous ABA in alleviating waterlogging induced-damages, thus, the development of an anti-waterlogging regulator by modifying ABA is a novel interesting project [6].

The role of JA-Me and its derivatives appeared in the last decades as a prominent signaling molecule implicated in stress defense and development in plants [58]. However, there is a very few experimental studies on the role of JA-Me in plant under waterlogging. Although, there are studies that have suggested that JA-Me and ARs formation are negatively correlated in *Arabidopsis* under waterlogging condition [59], [60]. The decrease of endogenous JA-Me was more marked in the leaves compared in adventitious roots in both species under waterlogging condition. Also, it was important to notice that during LT-WL the concentration of JA-Me in *C. operculatus* ARs did not decrease with WL + S-IAA, WL + W-IAA, WL + S-ABA, and WL + W-ABA treatments compared with single LT-WL treatment. The application of exogenous ABA enhanced massively the concentration of JA-Me in the ARs under LT-WL in the seedlings belong to *C. operculatus* which possess a stronger WL-tolerance. Our results suggest that the up-regulation of JA-Me in plants under waterlogging condition seems to play a major role in the accumulation ARs. Exogenous ABA or IAA could regulate positively JA-Me under LT-WL. Although this is one of the first reports that deal with the interactions among JA-Me accumulation, LT-WL, and exogenous plant hormones (IAA and ABA), further studies are needed to shed light on the key role of JA-Me in plants under submergence conditions. Furthermore, the results of the present study shed light on the networking of different signaling molecules, leading to physiological changes and waterlogging responses in the two tolerant waterlogging species. In conclusion, adventitious root formation and waterlogging tolerance in two tree species were complexes interactions among plant hormones, antioxidants, and photosynthetic parameters. The distribution of plant hormones in various plant organs under submergence conditions depended on the type of species and organ as well as on the time and space. The present study has showed a clear perception on the mechanism through which exogenous phytohormones (IAA and ABA) might alleviate LT-WL stress in woody plants, thus allow them to adapt to submergence conditions. Furthermore, the species that possesses larger morphological parameters (*C. operculatus*) had better waterlogging tolerance. WL + W-ABA promoted more WL-tolerance in both species in comparison to the other techniques. However, all exogenous phytohormones showed effectiveness to provide waterlogging-tolerance. Although, exogenous IAA promoted more ground biomass, exogenous ABA improved the below ground parameters.

## Materials and methods

### Plant material, growth, and waterlogging procedures

Two 6-month-old tree species, namely *C. operculatus* (strong waterlogging-tolerance) and *S. jambos* (medium waterlogging-tolerance), were obtained from a local commercial tree nursery in Guangzhou Zengcheng, China. Pot experiments using these trees were conducted in a greenhouse at Hainan University (20° 03′ 22.80“ N, 110° 19’ 10.20” E). The seedlings were transferred into plastic pots that were 21 cm in height and 19 cm in diameter and filled with substratum containing red soil, sand, and coconut coir (v: v: v = 4: 2: 1). The substratum was composed of 33.65 g kg^− 1^ organic carbon, 58.01 g kg^− 1^ organic matter, 1.77 g kg^− 1^ total nitrogen, and 0.64 g kg^− 1^ total phosphorus. The seedlings of *C. operculatus* and *S. jambos* were well-watered and grown for 2 months under natural conditions. One seedling per pot of *C. operculatus* and *S. jambos* was partly submerged for 120 d by upholding the water level at 10 cm above the soil surface.

### Experimental design

The seedlings of 4-month-old *C. operculatus* and *S. jambos* were subjected to different treatments. A complete randomized design was applied with two factors: (i) waterlogging (WL) and (ii) treatment with exogenous phytohormones (ABA and IAA). The experiment was conducted in six replicates, with four seedlings per replicate. Treatments were designed as follows: (1) CK: well-watered; (2) WL: WL treatment; (3) WL + S + IAA: WL treatment and IAA (0.5 mg/L) sprayed on the leaves; (4) WL + W + IAA: WL treatment and IAA (0.5 mg/L) watered in the substratum; (5) WL + S + ABA: WL treatment and ABA (1 μmol/L) sprayed on the leaves; and (6) WL + W + ABA: WL treatment and ABA (1 μmol/L) watered in the substratum. IAA and ABA were sprayed at 7 pm every 2 days in the front and back of *C. operculatus* and *S. jambos* leaves. For IAA or ABA watered treatment, all pots were immersed in aqueous solution containing 0.5 mg/L IAA (99% purity, Solarbio Co. Ltd., China) or 1 μmol/L ABA (Solarbio Co. Ltd., China). The water for WL, WL + W + IAA, and WL + W + ABA was replaced every 15 days. The concentration of ABA and IAA used in the present study was based on a previous research study [61].

### Growth analysis

After 120 days of treatment, samples were harvested and stored at − 80 °C for biochemical analysis. Plant height and blade number were measured or counted using the standard procedure. The harvested samples were split into primary roots (PRs), ARs, leaves, and stems at the harvest day. Fresh and dry biomass (shoot, ARs, PRs, and stem) was weighed. Total leaf area was measured by Portable Laser Area MeterLI-3000C (Li-Cor, Inc., United States).

### Photosynthesis analysis

The contents of photosynthetic pigments (Chl-a, Chl-b, Car, and T-Chlo) were measured according to a previously reported method [62]. Acetone solution (85%) was used to extract the pigment. Absorbance was read at 663, 646, and 470 nm. Gas exchange parameters including net photosynthetic rate (Pn), stomatal conductance (Gs), intercellular CO_2_ concentration (Ci), transpiration (Trr), and water use efficiency (Wue) were determined according to the method of Yang et al. (2010). Measurement was conducted using the LI-COR 6400 portable photosynthesis system (LI-COR Inc., USA) from 08:30 h to 11:30 h on intact expanded young leaves.

### Relative conductivity (RC), malondialdehyde (MDA), and superoxide activity (O_2_^-^)

Relative conductivity (RC) was measured following the method described in [63] with some modifications. Fully fresh leaves were cleaned with deionized water, and 10 discs from the leaves were incubated with 15 mL of deionized water. Conductivity (C1) was recorded after 2 h. The samples were then boiled for 45 min to determine (C2). RC was calculated using the following formula: RC (%) = C1/C2 × 100.

MDA level was determined following the method developed in [64]. Fresh samples were homogenized with 0.1% trichloroacetic acid (TCA) and centrifuged at 11000×g for 8 min. The supernatant (1 mL) was mixed with 4 mL of 20% TCA solution containing 0.5% thiobarbituric acid and incubated at 100 °C for 30 min. The reaction mixture was cooled on an ice bath and centrifuged at 11,000×g for 8 min. Absorbance was recorded first at 530 and corrected at 600 nm.

The amount of O_2_^•-^ produced was quantified by a modified method described in [65]. Fresh samples (0.15 g) were homogenized in 2 mL of phosphate buffer (pH 7.8) and centrifuged at 10000×g for 15 min. The supernatant was decanted and mixed with phosphate buffer. The reaction mixture included1 mL of the supernatant, 0.75 mL of 65 mM phosphate buffer, and 0.25 mL of 10 mM hydroxylamine hydrochloride. The mixture was incubated at 25 °C for 20 min and added to 1 mL of 17 mM sulfanilamide and 1 mL of 7 mM α-naphthylamine. The mixture was kept at 30 °C for 30 min, mixed with 4 mL of trichloromethane, and centrifuged at 10,000×g, 4 °C for 3 min. Absorbance was recorded at 530 nm by using NaNO_2_ as the standard curve to calculate O_2_^•-^activity.

### Proline and soluble proteins

Proline concentration was measured following the Bates’ method [66] with some modifications. About 200 mg of fresh leaf samples were mixed with 5 mL of 3% sulfosalicylic acid. The homogenate (1 mL) was added to 1 mL of acid-acetic ninhydrin reagent followed by 1 mL of glacial acetic acid. The mixture was then incubated at 100 °C for 1 h. The reaction was terminated by cooling the samples on ice water. Toluene was used to extract the chromophore-containing phase, and absorbance was recorded at 520 nm.

Soluble proteins were determined following the method described by Bradford [67] by using Coomassie Brilliant Blue G-250. About 2 mL of phosphate buffer (pH 7.8) was used as extraction solution. Fresh samples (100 mg) were mixed with phosphate buffer. Absorbance was read at 595 nm, and protein concentration was determined using a standard curve.

### Antioxidant enzymatic activities

Fresh leaf samples (0.2 g) were ground and homogenized with tissue grinder and scientific laboratory homogenizer (JXFSTPRP-24, Shanghai Jingxin Industrial Development Co., Ltd., China). The mixture contained liquid nitrogen, 5 mL of 50 mM sodium phosphate buffer (pH 7.8), and 1% polyvinylpolypyrrolidone (PVP). The mixture was centrifuged at 10,000 rpm and 4 °C for 15 min. The supernatant was used to determine antioxidant enzymatic activities.

Peroxidase (POD) activity was determined as described in [68] following the change of absorption at 470 nm caused by guaiacol oxidation. POD activity was expressed as unit• g^− 1^•FW•min^− 1^. Superoxide dismutase (SOD) activity was measured following the method described in [69] based on the inhibition of nitroblue tetrazolium chloride (NBT) reduction. Absorbance was recorded at 560 nm by using a spectrophotometer (UV-1800PC, Shanghai Mapada Instruments Co., Ltd., China). Catalase (CAT) was determined in accordance with the manufacturer’s instructions by using the method described in [70]. Phosphate buffer was provided in the assay kit from Nanjing Jiancheng Bioengineering Institute, China.

### Non-enzymatic antioxidant content

Reduced glutathione (GSH) and ascorbate acid (AsA) contents were measured according to the manufacturer’s instructions and the method in [68], [70]. About 100 mg of fresh leaves were ground in liquid nitrogen and homogenized with PBS (5 mg of fresh leaves to 50 μL of PBS). The mixture was centrifuged at 10,000 rpm and 4 °C for 30 min, and assay was performed with kits from Nanjing Jiancheng Bioengineering Institute, China.

### Phytohormone content

Fresh samples (leaves and ARs) were used to quantify the contents of endogenous plant hormones including IAA, ABA, JA-Me, and gibberellin (GA_3_). The extraction, purification, and quantification of plant hormones were conducted using ELISA methodology as described in [71]. Mouse monoclonal antigens and antibodies against the phytohormones were provided by the Phytohormones Research Institute (China Agricultural University).

### Fuzzy membership functions and principal component analysis

Subjective evaluation of the positive effect of various treatments on *C. operculatus* and *S. jambos* under WL was conducted based on Fuzzy membership functions as described previously [15]. The following equation was used: U (X*i*) = (X*i* - X*min*)/X*max*- X*min*, where U (X*i*) is the value of the subordinate function; X*i* is the parameter’s value; X*min* is the minimum value of this parameter; and X*max* is the maximum value. PCA and Pearson correlation among various parameters were performed with GraphPad prism 9.0.0. Canoco 5 (Microcomputer Power, Ithaca, USA) was used for elaborate PCA between treatments and parameters.

### Data analysis

All morphological, hormonal, physiological, and biochemical parameters were analyzed by two-way ANOVA using a factorial experiment in a completely randomized design. Tukey’s honest significant difference test was performed at *p* value of ≤0.05. GraphPad prism 9.0.0 was used to analyze data and draw graphs.

## Supplementary Information

**Additional file 1.**

**Additional file 2.**

**Additional file 3.**

**Additional file 4.**

**Additional file 5.**

**Additional file 6.**

## Data Availability

All data generated or analyzed during this study are included in this published article as supplementary excel files: file 1 (morphological raw data), file 2 (photosynthesis raw data), file 3 (physiological raw data) and file 4 (phytohormones raw data).
