# Supplementary figures and images for: Exogenous ABA and IAA modulate physiological and hormonal adaptation strategies in Cleistocalyx operculatus and Syzygium jambos under long-term waterlogging conditions
Source: BMC Plant Biol. 2022 Nov 10;22:523. doi: 10.1186/s12870-022-03888-z (PMC9648000; doi:10.1186/s12870-022-03888-z)

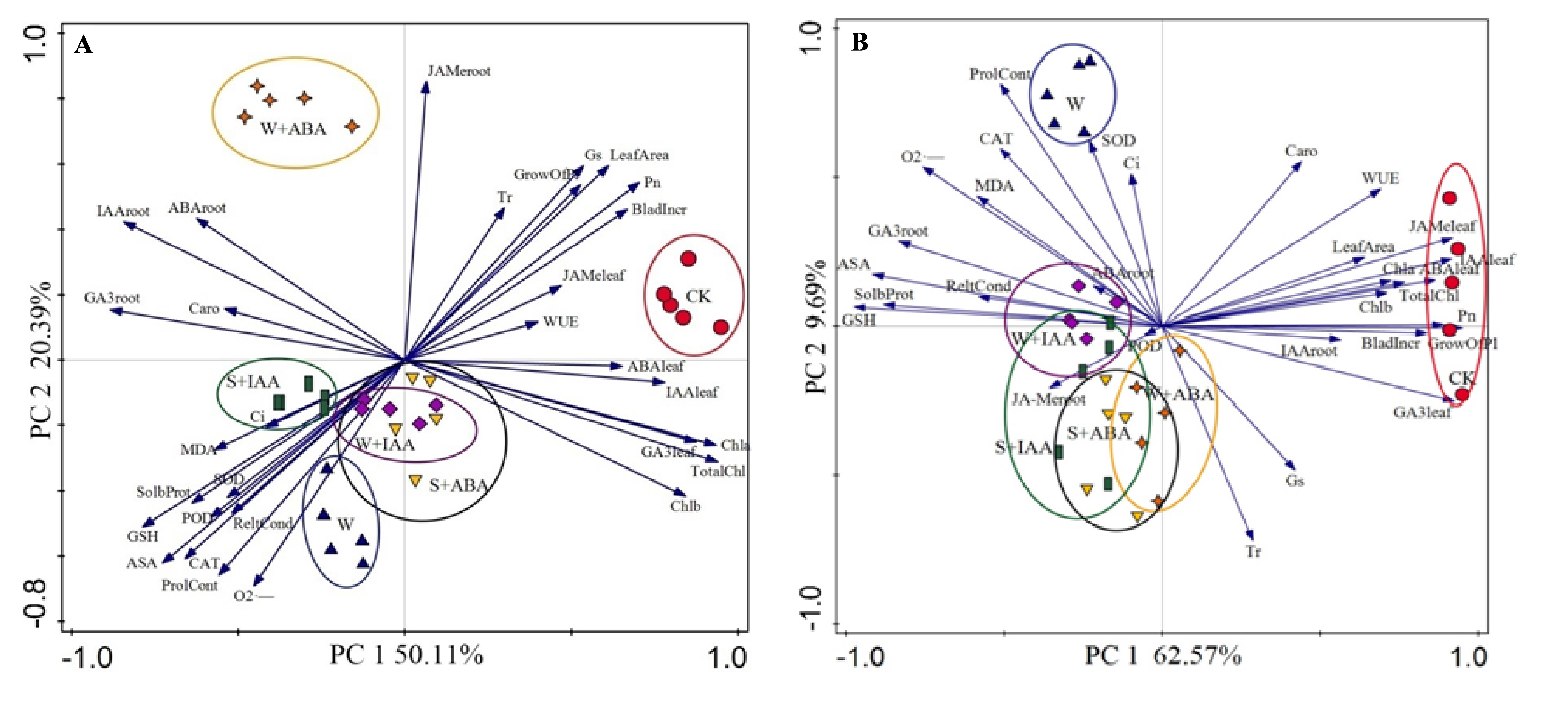

Supplement: Supplementary file 1 — Additional file 1. [file 12870_2022_3888_MOESM1_ESM.jpg]
