# Supplementary material for: Exogenous ABA and IAA modulate physiological and hormonal adaptation strategies in Cleistocalyx operculatus and Syzygium jambos under long-term waterlogging conditions
Source: BMC Plant Biol. 2022 Nov 10;22:523. doi: 10.1186/s12870-022-03888-z (PMC9648000; doi:10.1186/s12870-022-03888-z)
Supplement: Supplementary file 2 — Additional file 2. [file 12870_2022_3888_MOESM2_ESM.docx]

Supplementary Table 1: Subordinate function analysis involving all measured parameters in *C*. *operculatus* under LT-WL and exposed to IAA and ABA.

| Treatment | CK | W | S+IAA | W+IAA | S+ABA | W+ABA |
| --- | --- | --- | --- | --- | --- | --- |
| Plant height | 1.00 | 0.05 | 0.18 | 0.17 | 0.00 | 0.57 |
| Blades | 1.00 | 0.05 | 0.12 | 0.00 | 0.18 | 0.38 |
| Leaf area | 1.00 | 0.19 | 0.00 | 0.21 | 0.46 | 0.78 |
| Pn | 1.00 | 0.00 | 0.00 | 0.11 | 0.11 | 0.37 |
| Trr | 0.99 | 0.07 | 0.00 | 0.48 | 0.45 | 1.00 |
| Gs | 1.00 | 0.20 | 0.00 | 0.00 | 0.36 | 0.72 |
| Wue | 1.00 | 0.16 | 0.31 | 0.00 | 0.11 | 0.20 |
| Ci | 0.00 | 1.00 | 0.31 | 0.38 | 0.98 | 0.67 |
| Chl-*a* | 1.00 | 0.00 | 0.11 | 0.23 | 0.30 | 0.39 |
| Chl-*b* | 1.00 | 0.00 | 0.19 | 0.47 | 0.38 | 0.52 |
| T-Chlo | 1.00 | 0.00 | 0.13 | 0.29 | 0.32 | 0.42 |
| Caro | 0.00 | 1.00 | 0.73 | 0.77 | 0.35 | 0.89 |
| MDA | 1.00 | 0.00 | 0.24 | 0.13 | 0.06 | 0.29 |
| Proline content | 1.00 | 0.00 | 0.36 | 0.71 | 0.38 | 0.79 |
| Soluble protein | 1.00 | 0.00 | 0.45 | 0.53 | 0.45 | 0.53 |
| RC | 1.00 | 0.00 | 0.30 | 0.77 | 0.38 | 0.68 |
| ASA | 1.00 | 0.00 | 0.10 | 0.39 | 0.30 | 0.61 |
| GSH | 1.00 | 0.00 | 0.21 | 0.55 | 0.43 | 0.54 |
| O_2_^·-^ | 1.00 | 0.00 | 0.45 | 0.72 | 0.83 | 0.91 |
| CAT | 1.00 | 0.00 | 0.16 | 0.28 | 0.41 | 0.64 |
| POD | 1.00 | 0.00 | 0.26 | 0.77 | 0.14 | 0.63 |
| SOD | 1.00 | 0.00 | 0.38 | 0.45 | 0.30 | 0.52 |
| ABA in root | 0.00 | 0.50 | 0.26 | 0.03 | 0.35 | 1.00 |
| IAA in root | 0.00 | 0.40 | 0.50 | 0.20 | 0.29 | 1.00 |
| GA_3_ in root | 0.00 | 0.68 | 0.54 | 0.55 | 0.64 | 1.00 |
| JA-Me in root | 0.52 | 0.00 | 0.34 | 0.52 | 0.54 | 1.00 |
| ABA in leaf | 1.00 | 0.65 | 0.00 | 0.62 | 0.28 | 0.37 |
| IAA in leaf | 1.00 | 0.02 | 0.01 | 0.43 | 0.96 | 0.00 |
| GA_3_ in leaf | 1.00 | 0.32 | 0.30 | 0.34 | 0.53 | 0.00 |
| JA-Me leaf | 1.00 | 0.60 | 0.40 | 0.00 | 0.36 | 0.53 |
| Mean | **0.82** | **0.20** | **0.24** | **0.37** | **0.39** | **0.60** |

Supplementary Table 2: Subordinate function analysis involving all measured parameters in *S*. *jambos* under LT-WL and exposed to IAA and ABA.

| Treatment | CK | W | S+IAA | W+IAA | S+ABA | W+ABA |
| --- | --- | --- | --- | --- | --- | --- |
| Plant height | 1.00 | 0.04 | 0.16 | 0.00 | 0.09 | 0.20 |
| Blades | 1.00 | 0.17 | 0.23 | 0.00 | 0.11 | 0.43 |
| Leaf area | 1.00 | 0.23 | 0.20 | 0.50 | 0.00 | 0.20 |
| Pn | 1.00 | 0.08 | 0.00 | 0.05 | 0.27 | 0.10 |
| Trr | 0.94 | 0.00 | 0.96 | 0.67 | 0.91 | 1.00 |
| Gs | 1.00 | 0.00 | 0.62 | 0.54 | 0.77 | 0.54 |
| WUE | 1.00 | 0.47 | 0.00 | 0.16 | 0.31 | 0.09 |
| Ci | 0.40 | 1.00 | 0.08 | 0.79 | 0.00 | 0.70 |
| Chl-*a* | 1.00 | 0.11 | 0.16 | 0.46 | 0.00 | 0.40 |
| Chl-*b* | 1.00 | 0.19 | 0.31 | 0.33 | 0.00 | 0.48 |
| T-Chlo | 1.00 | 0.14 | 0.21 | 0.41 | 0.00 | 0.42 |
| Caro | 1.00 | 0.57 | 0.05 | 0.32 | 0.00 | 0.44 |
| MDA | 1.00 | 0.00 | 0.40 | 0.49 | 0.73 | 0.52 |
| Proline content | 1.00 | 0.00 | 0.79 | 0.46 | 0.97 | 0.91 |
| Soluble protein | 1.00 | 0.13 | 0.11 | 0.00 | 0.31 | 0.34 |
| RC | 1.00 | 0.00 | 0.36 | 0.31 | 0.14 | 0.48 |
| ASA | 1.00 | 0.00 | 0.27 | 0.30 | 0.30 | 0.39 |
| GSH | 1.00 | 0.00 | 0.24 | 0.21 | 0.23 | 0.37 |
| O_2_^·-^ | 1.00 | 0.00 | 0.53 | 0.60 | 0.69 | 0.77 |
| CAT | 1.00 | 0.00 | 0.65 | 0.77 | 0.75 | 0.78 |
| POD | 0.66 | 0.57 | 0.39 | 0.54 | 1.00 | 0.00 |
| SOD | 0.87 | 0.00 | 0.78 | 0.91 | 0.91 | 1.00 |
| ABA in root | 0.00 | 0.53 | 1.00 | 0.59 | 0.34 | 0.00 |
| IAA in root | 0.00 | 0.01 | 0.54 | 0.51 | 0.00 | 0.38 |
| GA3 in root | 0.00 | 1.00 | 0.93 | 0.52 | 0.42 | 0.43 |
| JA-Me in root | 0.00 | 0.17 | 1.00 | 0.15 | 0.01 | 0.32 |
| ABA in leaf | 1.00 | 0.30 | 0.06 | 0.00 | 0.37 | 0.24 |
| IAA in leaf | 1.00 | 0.18 | 0.00 | 0.06 | 0.20 | 0.08 |
| GA3 in leaf | 1.00 | 0.00 | 0.32 | 0.19 | 0.21 | 0.31 |
| JA-Me leaf | 1.00 | 0.20 | 0.10 | 0.07 | 0.00 | 0.10 |
| Mean | 0.83 | 0.20 | 0.38 | 0.36 | 0.33 | 0.41 |
